# A comprehensive meta-analysis and a case–control study give insights into genetic susceptibility of lung cancer and subgroups

**DOI:** 10.1038/s41598-021-92275-z

**Published:** 2021-07-16

**Authors:** Debmalya Sengupta, Souradeep Banerjee, Pramiti Mukhopadhyay, Ritabrata Mitra, Tamohan Chaudhuri, Abhijit Sarkar, Gautam Bhattacharjee, Somsubhra Nath, Susanta Roychoudhury, Samsiddhi Bhattacharjee, Mainak Sengupta

**Affiliations:** 1 Department of Genetics, University of Calcutta, 35, Ballygunge Circular Road, Kolkata, 700019 India; 2 Present Address: Greehey Children’s Cancer Research Institute, UT Health San Antonio, 8403 Floyd Curl Dr., San Antonio, TX-78229 USA; 3 Department of CHEST, IPGME&R, 244 A.J.C. Bose Road, Kolkata, 700020 India; 4 Saroj Gupta Cancer Centre and Research Institute, Mahatma Gandhi Road, Thakurpukur, Kolkata, 700063 India; 5 CSIR-Indian Institute of Chemical Biology, 4, Raja S.C. Mullick Road, Kolkata, 700032 India; 6 National Institute of Biomedical Genomics, Near Netaji Subhas Sanatorium Post Office, Kalyani, West Bengal 741251 India

**Keywords:** Statistical methods, Cancer genetics, Genetic predisposition to disease

## Abstract

Reports of genetic association of polymorphisms with lung cancer in the Indian subcontinent are often conflicting. To summarise and replicate published evidence for association with lung cancer and its subgroups. We performed a meta-analysis of candidate associations on lung cancer, its histological subtypes and smoking status in the Indian subcontinent following PRISMA guidelines. Multiple testing corrections were done by the Benjamini–Hochberg method through assessment of significance at a false discovery rate of 10%. We genotyped and investigated rs1048943/*CYP1A1* in a case–control sample from eastern India, followed by its global meta-analysis using a similar protocol. Meta-analysis of 18 variants of 11 genes reported in 39 studies (7630 cases and 8169 controls) showed significant association of rs1048943/*CYP1A1* [2.07(1.49–2.87)] and rs4646903/*CYP1A1* [1.48(1.93–1.95)] with overall lung cancer risk at 10% FDR, while nominal association (*p* < 0.05) was observed for del1/*GSTT1*, del2/*GSTM1*, rs1695/*GSTP1* and rs17037102/ *DKK2*. Subtype analysis showed a significant association of del1/*GSTT1* with adenocarcinoma, rs4646903/*CYP1A1* with squamous carcinoma, and rs1048943/*CYP1A1* with both. Association of rs4646903/*CYP1A1* in smokers and effect modification by meta-regression analysis was observed. Genotyping of rs1048943/*CYP1A1* that presented significant heterogeneity (*p* < 0.1) revealed an association with adenocarcinoma among eastern Indian smokers, while a global meta-analysis in 10458 cases and 10871 controls showed association with lung cancer and its subgroups. This study identified the susceptibility loci for lung cancer and its covariate-subgroups.

## Introduction

Despite several measures taken against tobacco smoking and consumption, lung cancer remains one of the leading causes of cancer-related mortalities worldwide, with a low 5-year survival rate^1^. Epidemiological data suggest that the global lung cancer burden has risen to 2.1 million new cases of all cancer cases and 1.8 million deaths, close to 1 in 5 cancer deaths^2^. A recent investigation has shown the increase in lung cancer incidence in the Indian subcontinent and East Asia^3^. Lung cancer incidences vary widely across geographical regions due to the admixture of different populations^4^. In India, lung cancer constitutes 5.9% of all new cancer cases and 8.1% of all cancer-related mortalities in both sexes^2^. The northeastern state of Mizoram accounts for the highest reported cases of lung cancer in both sexes^4^. Earlier reports stated that approximately one million of the total five million lung cancer deaths worldwide are contributed by India^5^, and the death toll is projected to rise to 1.5 million by 2020^5,6^. Smoking tobacco is considered the most significant factor in lung carcinogenesis^1,7^. Apart from tobacco smoking, betel quid chewing^8,9^, diet^10–12^, biofuel exposure^10–15^, asbestos exposure^10,11,16^ and other environmental pollutants^10,11,17,18^ contribute to lung carcinogenesis. Earlier studies have revealed a rise in lung cancer incidence among never smokers^19^, particularly in women of East Asian origin^20,21^.

Genome-wide association studies (GWAS) in the Chinese population have identified 16 susceptibility loci (p ≤ 5.00 × 10^−8^) associated with lung cancer risk^22,23^, and 4 loci out of them showed evidence of association with lung cancer risk in smokers^22^. Similarly, another GWAS on subjects of European ancestry with 29,266 lung cancer patients and 56,450 controls identified 18 susceptibility loci (*p* ≤ 5.00 × 10^−8^), including 10 novel loci^21^. Interestingly, the association of the 10 novel loci varied across different histological subtypes. Out of the 10 loci, four were associated with overall lung cancer risk, while the remaining 6 loci were associated with lung adenocarcinoma^21^. Most of the GWAS was done on European or Chinese descent subjects, and the majority of the identified risk alleles have not been evaluated in the population of the Indian subcontinent despite several candidate gene association studies^24–33^. Contradictory outcomes of case–control association studies of the same polymorphism by different authors failed to identify the genes' overall effect and the genetic variations on lung cancer susceptibility in the region. The differences in genetic association across the geographical regions of the Indian subcontinent, comprised of distinct population groups, might be attributed to gene–gene and gene-environment interactions, which could act as potential modulators of lung cancer risk^34^. Contradictory study results can be due to small sample sizes, heterogeneity between study samples and racial/ethnic differences of the source populations^35^ within the Indian subcontinent.

Further, the differences in socio-economic and cultural practices in different parts of the Indian subcontinent might contribute to diverse lifestyle habits like smoking, chewing of tobacco and betel quids, alcohol consumption, and exposure to air pollutants; exposure to asbestos and other occupational hazards that in turn could modify the risk of the disease. This brings forth the importance of meta-analysis, a robust statistical method^36^, to assess the variant(s) pooled effect on lung cancer susceptibility in the concerned population by pooling the individual study data.

The present investigation aimed to estimate the pooled association measure of reported candidate genetic variants for the Indian subcontinent through a workflow (Supplementary Information, Fig. S1) as in our previous study^37^. Some results of the meta-analysis were further investigated in an independent case–control sample. Significantly associated variants were compared to other populations and ethnicities worldwide (Supplementary Information, Fig. S2).

## Methods

The scheme of analysis followed in this study is explained and summarised in (Supplementary Information, Fig. S2).

### Identification and eligibility of studies

The current study followed the PRISMA guidelines^38^. Systematic mining of the databases, such as PubMed, Scopus, and Web of Science, was done to select appropriate studies using the following keywords: (SNP/SNPs/polymorphisms/single nucleotide polymorphisms/SNVs/SNV/Mutation/ Variants/Genotypes/Alleles); (Lung cancer/Lung Carcinoma/Lung malignancy/Lung neoplasm); (India/Pakistan/Nepal/Bangladesh /Bhutan/Sri-Lanka/Maldives/Afghanistan). The eligibility of all the identified case–control studies on lung cancer was curated and selected manually by two authors and rechecked by the other authors. Hardy–Weinberg Equilibrium (HWE) was assessed in the controls by *goodness-of-fit* chi-square test (*p* < *0.05*) for all the variants using the R package ‘genetics’^39^. We could not assess HWE for the deletion polymorphisms of *GSTT1* and *GSTM1*, which was presented in the selected studies without the heterozygous genotype counts.

### Inclusion and exclusion criteria

The selection of the studies for meta-analysis was made following the specific inclusion criteria: (a) samples should be from populations belonging to the countries of the Indian subcontinent; (b) genotype counts of cases and controls need to be reported for each investigated genomic variant (c) only full research article of original studies were included (d) all association studies published till 31^st^ December 2019 were considered (e) studies should be published in English.

The exclusion criteria were as follows: (a’) duplicated studies using the same population (b’) the studies inconsistent with Hardy–Weinberg Equilibrium (HWE). However, the variants reported in at least three independent studies on different sample sets were considered for this study.

### Data extraction

Data extraction from the literature was done following specific inclusion and exclusion criteria. The data collected from the selected studies are (1) first author surname, (2) year of the publication, (3) mean age with standard deviation, (4) sex, (5) smoking status, (6) histological types, (7) genetic polymorphisms and (8) genotype-specific case–control data (9) geographical region of sampling done in the selected studies.

Genotype counts of the lung cancer cases and controls were collected for all 18 variants. The cases' genotype counts were stratified within the histological subtypes of lung cancer for del1/*GSTT1*, del2/*GSTM1*, rs4646903/*CYP1A1*, and rs1048943/*CYP1A1* only. The remaining variants lacked the histological subtype-stratified genotype counts for the cases and were not included in the analysis. For the variants del1/*GSTT1*, del2/*GSTM1*, rs4646903/*CYP1A1*, and rs1048943/*CYP1A1,* we looked for smoking status-stratified genotype counts as described earlier^37,40^.

### Study-level summary estimates and selection of genetic model

Logistic regression of lung cancer status on variant genotype was done using additive, recessive and dominant effect models (using R function ‘glm’) to obtain the study-level unadjusted odds ratio (OR), standard errors (SE) and 95% confidence intervals (95% CI). Adjustment for covariates, such as smoking and sex, could not be done as some of the selected studies did not present sufficient data.

Apart from del1/*GSTT1*, del2/*GSTM1*, rs1048943/*CYP1A1* and rs4646903/*CYP1A1*, the remaining 14 polymorphic variants were analysed in 3 different genetic models: i.e. additive, dominant and recessive models as we did not know which model will give better outcomes. The selection of the genetic models for the four variants in our analysis was done based on the models used in the respective studies. Thus, the variants del1/*GSTT1* and del2/*GSTM1* were analysed in the recessive model, while rs1048943/*CYP1A1* and rs4646903/*CYP1A1* were only analysed in a dominant model.

### Meta-analysis

Meta-analysis was conducted in R software^41^ package ‘metafor’^42^ on lung cancer genetic association reports from the Indian subcontinent. Both fixed-effect (FE) meta-analysis (inverse-variance weighting) and random-effects (RE) meta-analysis (DerSimonian Laird method) were used to combine the study-level estimates (using the ‘rma.uni’ function in R package ‘metafor’^42^). It estimates cumulative odds ratios and 95% confidence intervals (95% CI) to determine the overall evidence of statistical association (*p* < 0.05) of the reported variants with lung cancer risk. Benjamini–Hochberg method was used to correct multiple testing, assessing significance at a false discovery rate (FDR) less than 10% level (*p*_*FDR*_ < *0.1*). The selection of variants was made based on the *p-values* of the FE meta-analysis. However, for variants with significant heterogeneity, the summary estimates from the RE model are more reliable^43^. Inter-study heterogeneity was evaluated using Cochran’s Q test (*p*_*Het*_ < *0.1*)^44^ and heterogeneity index (I^2^)^45–48^.

### Effect of histological subtypes and smoking

For histological subtypes and smoking, we performed subgroup stratified meta-analyses. First, we generated study-level summary data using logistic regression (generalised linear model; ‘glm’) of disease status on subgroup-specific genotype counts. We performed the meta-analysis within each subgroup using the methods described earlier. Finally, we performed a fixed-effect meta-regression to test for effect modification (interaction) by smoking status. For this, we used stratum (i.e., smoker/non-smoker group) as a moderator variable using the ‘rma.uni’ function in R package ‘metafor’.

### Publication bias

A visual inspection of funnel plots^49^ for variants reported in more than five different studies along with Egger’s regression test for variants reported in more than ten different studies was done to evaluate the asymmetry (*p* < 0.05) of the funnel plots for the estimation of publication bias, if any, among the selected studies.

### Case–control study on the East Indian population

Lung cancer patients (N = 101) with a recent history of tobacco smoking, including males and females, of all the histological subtypes, were recruited from the Saroj Gupta Cancer Centre and Research Institute (SGCCR&I) and the Department of CHEST, IPGME&R in Kolkata. Individuals who had quit smoking ≥ 15 years from the date of recruitment were excluded. Clinico-radiologically confirmed healthy smokers, aged ≥ 55 years^2^, following NCCN guidelines^50^, without any history of cancer but from the same geographical region were recruited as controls (N = 413). All study participants were asked to provide their informed consent for voluntary participation before sample collection, following the concerned institutes' ethical guidelines and the Declaration of Helsinki, 1964. A detailed questionnaire was filled up under medical supervision with the clinical data.

We conducted a case–control association of rs1048943/*CYP1A1* with lung cancer among smokers in the East Indian population, including 101 cases and 413controls. The polymorphism was selected from the current meta-analysis on lung cancer as the significant polymorphic variant after FDR correction with significant heterogeneity between the studies.

#### Genotyping

The PCR–RFLP technique was used for genotyping of rs1048943 (CYP1A1). The primer sequences used for the PCR of the fragment of 204 base pairs harbouring the polymorphism rs1048943 of *CYP1A1* were as follows: *CYP1A1*-F: 5’-CTGTCTCCCTCTGGTTAC AGGAAGC-3’, and *CYP1A1*-R: 5’-TTCCACCCGTTGCAGCAGG ATAGC-3’. The PCR conditions followed for adequate amplification were as follows: 94 °C/5 min–(94 °C/40 s–61 °C/40 s–2 °C/40 s) × 30 cycles–72 °C/7 min–4 °C hold. Following PCR, a quality check of the amplicons was done in 6% Polyacrylamide gel electrophoresis (PAGE). The BsrDI restriction enzyme digested the PCR amplicons at 65 °C for 2 h. We performed logistic regression of the lung cancer status on the genotype counts using R v3.4.2 software. Covariate-adjusted analysis was done for age, sex, ethnicity, smoking intensity in pack-years, alcohol consumption, tobacco and betel quid chewing, and asbestos exposure. We assessed Hardy–Weinberg Equilibrium (HWE) in the genotyped controls by *goodness-of-fit* chi-square test (*p* < *0.05*) using the R package ‘genetics’^39^.

### Meta-analysis of significant variants in the global population

We performed a meta-analysis including all the reported populations worldwide for the lung cancer-associated non-synonymous variant after Benjamini–Hochberg FDR correction in the Indian subcontinent that showed heterogeneity, following the same protocol.

### Ethics approval

The Ethics Committee of Saroj Gupta Cancer Centre and Research Institute (IEC SGCCRI Ref No- 2017/MS/1; dated: 11.10.2017), IPGME&R (Memo No. Inst/IEC/2015/545; dated: 10.12.2015), Kolkata and the University of Calcutta (Ref No: 0024/16–117/1434; dated: 24.10.2016), Kolkata, India; approved the study with human subjects as per the regulation of the Indian Council of Medical Research (ICMR) following the Declaration of Helsinki, 1964.

### Consent to participate

Informed consent was obtained from all individual participants included in the study.

### Consent for publication

The human participants have consented to the submission of the case report to the journal.

## Results

### Study characteristics

Systematic mining of the databases with the search strings mentioned above revealed 1060 hits, screened down to 39 studies following the specific inclusion/exclusion criteria set for the proposed study (Supplementary Information, Fig.S3). These 39 studies included 18 polymorphisms from 11 genes with 7,630 cases and 8,169 controls (Table 1). Covariate specific case–control data, particularly tobacco smoking, mean age, histological status, and geographical region of the subjects, were recorded from all 39 studies selected for meta-analysis (Supplementary Information, Table S1).

**Table 1:** Details of the selected studies for meta-analysis.

Selected studies	PMID	Number of cases/controls	Mean age ± SD cases	Mean age ± SD controls	Sex of cases	Sex of Controls	Number of Smokers in Cases/ Controls	Zone of the Indian subcontinent
Bag et al.2014	25,097,401	26/33	56.11 ± 9.22	28.68 ± 6.77	24 M, 2F	33 M	(23/08)	North
Shukla et al.2013	23,803,127	218/238	56.14 ± 11.91	56.15 ± 7.84	189 M,29F	191 M,47F	128/34	North
Sobti et al.2004	15,646,021	100/76	55.5 ± 11.3	50.9 ± 8.1	95 M,5F	73 M,3F	86/59	North
Sharma et al.2015	26,529,288	270/270	57.39 ± 10.60	53.23 ± 10.49	235 M,35F	233 M,37F	212/193	North
Kumar et al.2009	19,009,239	93/253	42.6 ± 6.3	39.8 ± 5.4	81 M,12F	203 M,50F	81/101	North
Ihsan et al.2014	25,027,082	154/154	59.16 ± 9.95	60.39 ± 10.43	38 M,116F	38 M,116F	105/71	East
Phukan et al.2014	24,815,479	230/460	59.01 ± 13.02	58.60 ± 13.99	230F	460F	126/153	East
Ihsan et al.2011	22,206,016	188/209	60.41 ± 10.58	57.19 ± 10.75	145 M,43F	159 M,50F	132/139	East
Sreeja et al.2008	18,472,644	111/111	57.82 ± 11.74	56.21 ± 10.36	82 M,29F	84 M,27F	44/19	South
Peddireddy et al.2016	27,090,234	246/250	57.57 ± 10.19	58.06 ± 9.56	177 M,69F	180 M,70F	106/63	South
Sreeja et al.2005	16,228,113	146/146	58.17 ± 10.95	56.06 ± 10.67	133 M,13F	128 M,18F	102/62	South
Sobti et al.2008	18,415,801	151/151	56.9 ± 10.4	56.4 ± 11.1	130 M,21F	123 M,28F	123/112	North
Shah et al.2008(a)	18,573,508	200/200	56 ± 9	43 ± 12	200 M	200 M	120/62	North
Singh et al.2010	22,072,123	200/200	53 ± 10	51 ± 09	200 M	200 M	117/64	North
Saikia et al.2014	24,716,924	272/544	61.96 ± 11.91	61.79 ± 12.21	130 M,142F	260 M,284F	197/341	East
Natukula et al.2013	24,175,813	100/101	56.14	48.07	73 M,27F	86 M,15F	51/36	South
Uppal et al.2014	25,584,213	100/100	63.70 ± 3.91	62.08 ± 2.88	64 M ,36 F	76 M, 24F	76/56	North
Sreeja et al.2008(a)	17,952,468	211/211	57.82 ± 11.74	56.21 ± 10.36	182 M,29F	184 M,27F	143/119	South
Pachouri et al.2007	17,417,947	103/122	Not Reported	Not Reported	90 M,13F	104 M,18F	80/28	North
Sobti et al.2009	19,558,213	151/151	56.9 ± 0.4	56.4 ± 1.1	130 M, 21F	123 M,28F	123/112	North
Sobti et al.2003	14,602,525	100/76	55.59 ± 11.3	50.99 ± 8.1	95 M,5F	73 M,3F	86/59	North
Ihsan et al.2011(a)	21,043,833	161/274	60.24 ± 10.77	53.21 ± 13.37	120 M, 41F	202 M,72F	110/139	East
Jain et al.2005	16,354,872	40/40	Not Reported	Not Reported	35 M, 5F	35 M, 5F	32/32	North
Kumari et al.2016	27,614,750	420/420	57.87 ± 10.39	52.14 ± 10.9	359 M, 61F	353 M, 67F	345/303	North
Singh et al.2016	27,707,541	330/325	57.9 ± 10.58	52.27 ± 10.84	285 M, 45F	265 M, 60F	268/223	North
Tilak et al.2013	23,317,414	175/202	56.5 ± 10.3	54.99 ± 8.1	169 M, 06F	191 M, 11F	146/159	North
Girdhar et al.2016	27,396,354	353/351	57.55 ± 10.69	52.84 ± 10.80	305 M,48F	300 M,51F	278/250	North
Shah et al.2008(b)	18,082,227	294/263	56 ± 9	43 ± 12	200 M,0F	200 M,0F	120/62	North
Shaffi et al.2009	19,827,888	190/248	52.7	53.2	87 M,22F	95 M,68F	84/98	North
Lawania et al.2017	29,035,087	370/370	58.11 ± 10.44	53.83 ± 10.18	319 M,51F	319 M,51F	303/271	North
Bahl et al.2017(a)	27,640,551	300/300	57.38 ± 10.74	53.23 ± 10.44	258 M,42F	253 M,47F	235/208	North
Bahl et al.2017(b)	28,378,643	303/305	57.38 ± 10.74	53.23 ± 10.44	262 M,41F	269 M,36F	239/223	North
Bahl et al.2018	30,346,805	181/31	57.87 ± 10.81	Not Reported	184 M,28F	Not Reported	171/0	North
Singh et al.2017	28,332,164	193/48	58.71 ± 10.38	0	215 M,26F	0	200/0	North
Bhardwaj et al. 2017	29,412,865	250/237	57.38 ± 10.74	53.23 ± 10.44	252 M,40F	241 M,22F	229/197	North
Bahl et al.2017(c)	29,749,862	292/263	57.38 ± 10.74	53.23 ± 10.44	252 M,40F	241 M,22F	229/197	North
Chowdhury et al.2015	25,921,167	50/50	55 ± 10	46 ± 11	40 M, 10F	39 M, 11F	44/Not Reported	East
Islam et al.2013	23,178,447	106/116	57.87 ± 10.12	58.14 ± 9.77	93 M, 13F	105 M, 11F	97/104	East
Masood et al.2016	27,461,642	252/270	54 ± 11.5	53 ± 12.6	184 M, 68F	197 M, 73F	131/78	North

### Meta-analysis for the association of polymorphic variants with lung cancer and its subgroups in the Indian Subcontinent

In the FE meta-analysis of the 18 variants in the Indian subcontinent, we found six variants to be associated with overall lung cancer risk at nominal significance (*p* < 0.05), as shown in (Table 2). It included rs1048943/*CYP1A1*, rs1695/*GSTP1* and rs4646903/*CYP1A1* in the dominant model, del1/*GSTT1* and del2/*GSTM1* in the recessive model and rs17037102/*DKK2* in both additive and dominant models. After Benjamini-Höchberg false discovery rate (FDR) correction, two variants (rs1048943 and rs4646903) were significant at 10% FDR in the dominant model only. Out of these six nominal associations, two variants (rs1695 and rs1048943) showed significant heterogeneity (*p*_*Het*_ < *0.1*) by Cochran’s Q test. Hence, summary estimates of RE meta-analysis are also tabulated for these variants (Table 2). Additional smoking and histology-stratified analyses were carried out for the nominal associations. The detailed results are summarised below:

**Table 2:** A comprehensive list of meta-analysis results showing the overall association of the variants with lung cancer, with crude odds ratio (OR), 95% Confidence Interval (CI), p_FDR_, Benjamini-Höchberg False Discovery Rate (FDR) corrected p-value, Heterogeneity indices H^2^, I^2^. Both the Genetic model and model used for meta-analysis are also mentioned.

Gene Symbol	Variant ID	Number	Test for Association				Heterogeneity		
			Comparison	Model	OR (95% CI); *p-value*^†^	*p*_*FDR*_^‡^	H^2^	*p*_*Het*_^§^	I^2^
(A) Dominant Model									
***GSTP1***	**rs1695**	**3**	**(GG + AG) *vs* AA**	**Fixed**	**1.84 (1.07–3.16); *0.03***	*0.15*	5.02	***0.007***	80.08
				Random	1.88 (0.56–6.38); *0.31*	—			
*TP53*	rs1042522	6	(CC + CG) *vs* GG	Fixed	1.12 (0.75–1.68); *0.58*	*0.84*	2.62	*0.15*	61.82
***CYP1A1***	**rs4646903**	**11**	**(TC + CC) vs TT**	**Fixed**	**1.48 (1.93–1.95); *0.005***	***0.07***	1.19	*0.84*	15.98
***CYP1A1***	**rs1048943**	**8**	**(AG + GG) vs AA**	**Fixed**	**2.07 (1.49–2.87); *0.00002***	***0.07***	2.17	***0.06***	53.92
				**Random**	**2.03 (1.23–3.30); *0.004***	—			
*ERCC2*	rs13181	3	(CC + CA) *vs* AA	Fixed	1.32 (0.84–2.08); *0.23*	*0.49*	1.07	*0.71*	6.52
*DKK3*	rs3206824	3	(AA + AG) vs GG	Fixed	1.13 (0.66–1.93); *0.65*	*0.71*	1.04	*0.8*	3.68
*XRCC1*	rs25487	8	(AA + AG) vs GG	Fixed	0.94 (0.68–1.31); *0.73*	*0.71*	2.19	*0.102*	54.4
*XRCC1*	rs1799782	4	(TT + CT) vs CC	Fixed	1.2 (0.73–1.97); *0.47*	*0.7*	1.05	*0.86*	4.83
*XRCC1*	rs915927	3	(GG + AG) vs AA	Fixed	1.12 (0.68–1.87); *0.65*	*0.71*	1	*0.94*	0.22
*DKK2*	rs447372	3	(GG + AG) *vs* AA	Fixed	0.73 (0.44–1.22); *0.23*	*0.49*	1.05	*0.75*	3.52
*DKK2*	rs419558	3	(TT + TC) *vs* CC	Fixed	1.47 (0.85–2.55); *0.17*	*0.61*	1.61	*0.34*	37.79
***DKK2***	**rs17037102**	**3**	**(AA + AG) *vs* GG**	**Fixed**	**1.82 (1.03–3.22); *0.04***	*0.16*	1	*0.99*	0.004
*AXIN 2*	rs2240308	3	(TT + TC) *vs* CC	Fixed	0.79 (0.47–1.36); *0.41*	*0.68*	1.03	*0.83*	2.89
*AXIN 2*	rs2240307	3	(CC + CT) *vs* TT	Fixed	0.59 (0.28–1.24); *0.17*	*0.49*	1.01	*0.93*	0.56
*AXIN 2*	rs35285779	3	(GG + AG) vs AA	Fixed	0.87 (0.48–1.57); 0.64	*0.71*	1	*0.98*	0.02
*AXIN 2*	rs9915936	3	(AA + AG) vs GG	Fixed	0.63 (0.31–1.26); 0.19	*0.49*	1.13	*0.64*	11.83
(B) Recessive Model									
***GSTT1***	**del 1**	**14**	**Null (-/-) *vs* Present [(+ /-) + (+ / +)]**	**Fixed**	**1.36 (1.03–1.79); *0.028***	*0.42*	1.45	*0.49*	31.1
***GSTM1***	**del 2**	**15**	**Null (-/-) *vs* Present [(+ /-) + (+ / +)]**	**Fixed**	**1.38 (1.09–1.75); *0.008***	*0.42*	1.88	*0.29*	46.9
*GSTP1*	rs1695	3	GG *vs* (AG + AA)	Fixed	1.14 (0.52–2.48); *0.74*	*0.99*	1.45	*0.34*	30.97
*TP53*	rs1042522	6	CC *vs* (CG + GG)	Fixed	1.15 (0.75–1.76); *0.52*	*0.94*	1.77	*0.37*	43.62
*ERCC2*	rs13181	3	CC *vs* (CA + AA)	Fixed	1.49 (0.82–2.71); *0.19*	*0.77*	1.05	*0.73*	5.06
*DKK3*	rs3206824	3	AA vs (AG + GG)	Fixed	1.27 (0.49–3.27); *0.62*	*0.94*	1.01	*0.88*	1.18
*XRCC1*	rs25487	8	AA vs (AG + GG)	Fixed	1.15 (0.79–1.67); *0.48*	*0.94*	1.14	*0.83*	12.32
*XRCC1*	rs1799782	4	TT vs (CT + CC)	Fixed	1.07 (0.47–2.42); *0.88*	*0.99*	1.6	*0.4*	37.54
*XRCC1*	rs915927	3	GG vs (AG + AA)	Fixed	0.88 (0.46–1.69); *0.71*	*0.94*	1	*0.95*	0.17
*DKK2*	rs447372	3	GG *vs* (AG + AA)	Fixed	0.77 (0.39–1.51); *0.45*	*0.94*	1.64	*0.25*	38.85
*AXIN 2*	rs2240308	3	TT *vs* (TC + CC)	Fixed	0.72 (0.41–1.26); *0.25*	*0.77*	1.03	*0.81*	3.09
(C) Additive Model									
*GSTP1*	rs1695	3	GG *vs* AG *vs* AA	Fixed	1.13 (0.71–1.81); *0.61*	*0.88*	1.18	*0.56*	15.26
*TP53*	rs1042522	6	CC *vs* CG *vs* GG	Fixed	1.09 (0.79–1.52); *0.58*	*0.89*	2.1	*0.28*	52.46
*ERCC2*	rs13181	3	CC *vs* CA *vs* AA	Fixed	1.28 (0.86–1.90); *0.23*	*0.72*	1.02	*0.86*	1.6
*DKK3*	rs3206824	3	AA vs AG vs GG	Fixed	1.13 (0.68–1.87); *0.64*	*0.88*	1.03	*0.82*	2.96
*XRCC1*	rs25487	8	AA vs AG vs GG	Fixed	0.99 (0.76–1.32); *0.99*	*0.93*	1.35	*0.59*	25.99
*XRCC1*	rs1799782	4	TT vs CT vs CC	Fixed	1.16 (0.74–1.84); *0.52*	*0.88*	1.09	*0.79*	8.59
*XRCC1*	rs915927	3	GG vs AG vs AA	Fixed	1.04 (0.67–1.62); *0.86*	*0.93*	1	*0.99*	0.01
*DKK2*	rs447372	3	GG *vs* AG *vs* AA	Fixed	0.77 (0.50–1.23); *0.29*	*0.72*	1.09	*0.63*	8.12
*DKK2*	rs419558	3	TT *vs* TC *vs* CC	Fixed	1.45 (0.85–2.55); *0.17*	*0.72*	1.59	*0.35*	37
***DKK2***	**rs17037102**	**3**	**AA *vs* AG *vs* GG**	**Fixed**	**1.81 (1.03–3.15); *0.04***	*0.53*	1	*0.99*	0.00002
*AXIN 2*	rs2240308	3	TT *vs* TC *vs* CC	Fixed	0.82 (0.53–1.25); *0.35*	*0.72*	1.02	*0.85*	2.3
*AXIN 2*	rs2240307	3	CC *vs* CT *vs* TT	Fixed	0.66 (0.33–1.33); *0.25*	*0.72*	1.01	*0.92*	0.56
*AXIN 2*	rs35285779	3	GG vs AG vs AA	Fixed	0.89 (0.50–1.58); 0.69	*0.88*	1	*0.97*	0.08
*AXIN 2*	rs9915936	3	AA vs AG vs GG	Fixed	0.66 (0.33–1.29); 0.22	*0.72*	1.23	*0.55*	18.38

^†^p-value < 0.05, OR, Crude Odds Ratio, 95% CI, 95% Confidence Interval, I^2^ and H^2^ are measures of heterogeneity, ^‡^p_FDR_ < 0.1. ^§^p_Het_ < 0.1 (Cochran’s Q test), Significant associations are depicted in bold. N/A stands for Not Applicable.

#### CYP1A1/rs1048943 (dominant model)

**Figure 1:**
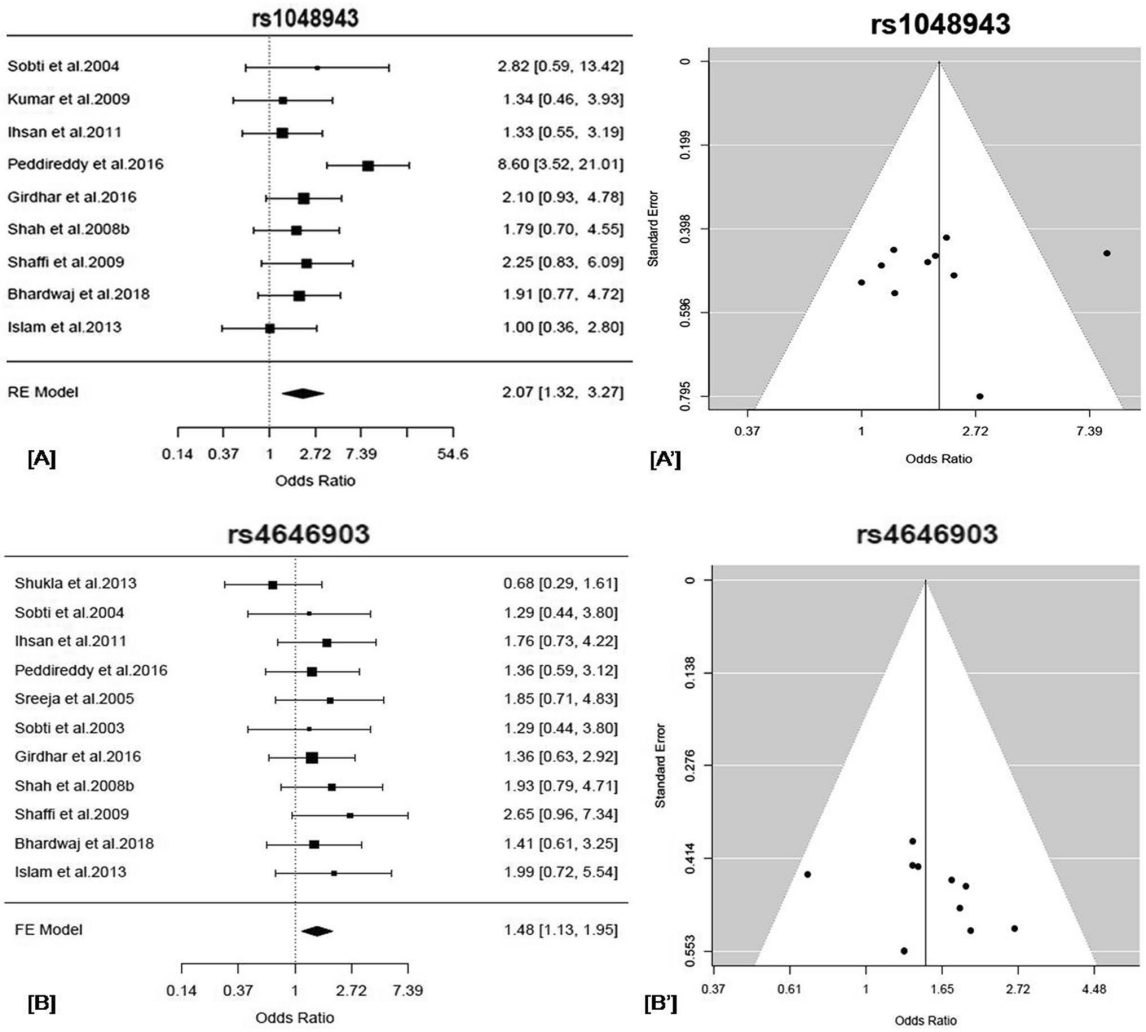
(**A**) Forest plot depicting the odds ratios (ORs), and 95% CI of the polymorphism, rs1048943/*CYP1A1* for its association with overall lung cancer risk in the Indian subcontinent in the random-effects model, (**A’**) Funnel plot that shows no evidence of publication bias between the studies reporting the polymorphism, rs1048943/*CYP1A1*. (**B**) Forest plot depicting the odds ratios (ORs) and 95% CI of the polymorphism, rs4646903/ *CYP1A1, *is associated with overall lung cancer risk in the Indian subcontinent in a fixed-effect model. (**B’**) Funnel plot shows no evidence of publication bias between the studies reporting the polymorphism, rs4646903/*CYP1A1*. The results are obtained in a dominant model of analysis. The forest plots of the significant associations were given (*p* < 0.05*). The figures were generated in the ‘metafor’ package (http://www.metafor-project.org) of R software (https://cran.r-project.org/).

Meta-analysis using FE model, we found a significant association of the variant with overall lung cancer risk (AG + GG *vs.* AA: OR = 2.07, 95% CI = 1.49–2.87, *p* = 0.00002, *p*_*FDR*_ = 0.07, Q^2^ = 2.17, *p*_*Het*_ = 0.06, I^2^ = 53.92%) (Table 2). After multiple testing adjustment by Benjamini–Hochberg FDR correction, the variant showed significant association with lung cancer (*p*_*FDR*_ = 0.07). Significant heterogeneity was observed for the variant rs1048943. The RE estimates also showed nominal association of the variant with lung cancer (AG + GG *vs.* AA: OR = 2.03, 95% CI = 1.23–3.30, *p* = 0.004) (Table2 and Fig. 1). No publication bias was observed from the inspection of the funnel plots (Fig. 1).

Interestingly for the variant rs1048943/*CYP1A1*, most of the signal was driven by a single study^31^, which could be the reason behind the observed heterogeneity between studies. Meta-analysis performed after excluding the study revealed a nominal association of rs1048943/*CYP1A1* [AG + GG *vs* AA: OR = 1.67, 95% C.I. = 1.18─2.25; *p* = *0.003*] with overall lung cancer without any significant heterogeneity.

#### Association with lung cancer histological subtypes

**Figure 2:**
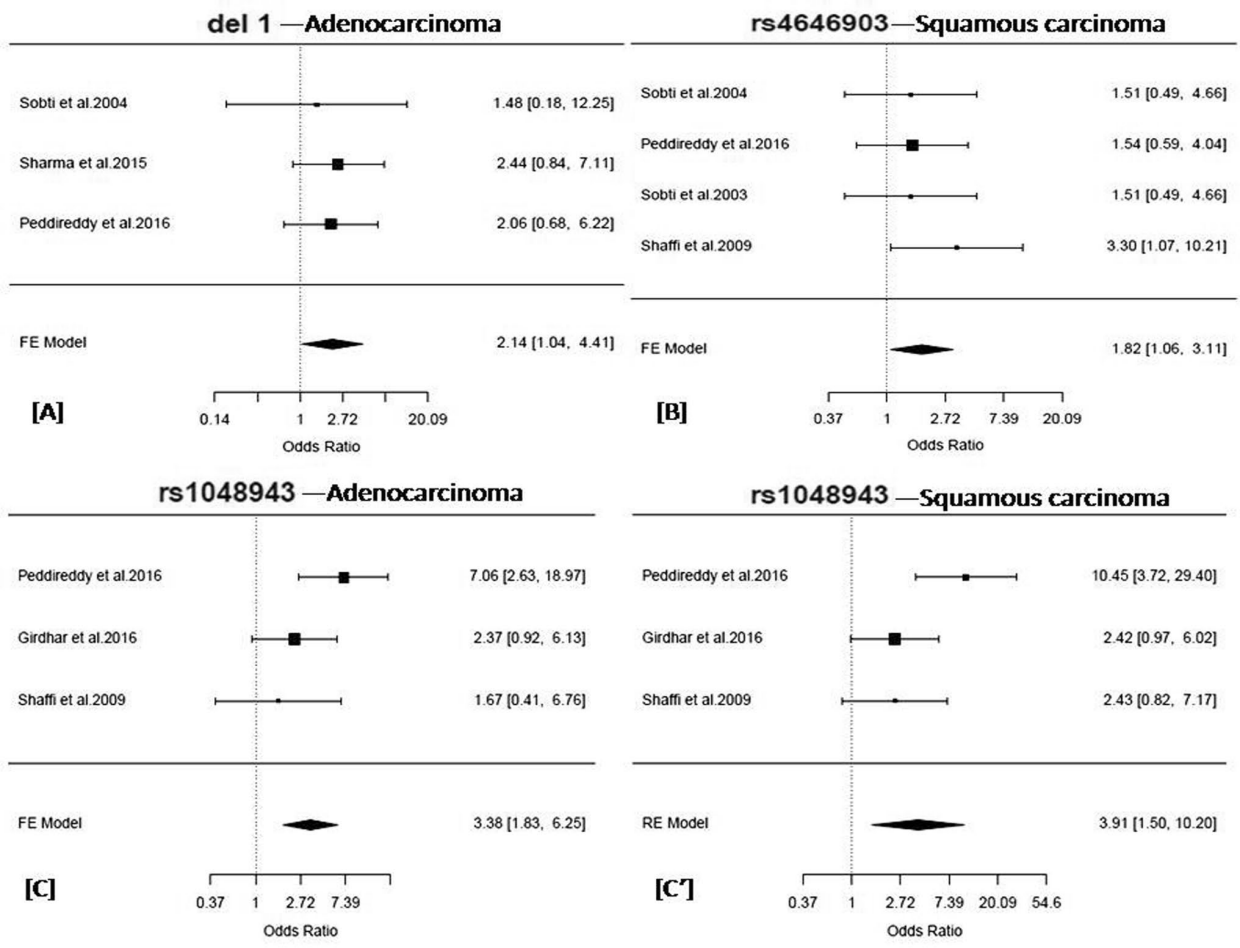
(**A**) Forest plot depicting the odds ratios (ORs), and 95% CI of the polymorphism, del1/*GSTT1* for its association with lung adenocarcinoma in a recessive model in the Indian subcontinent, (**B**) Forest plot depicting the odds ratios (ORs), and 95% CI of the polymorphism, rs4646903/*CYP1A1* for its association with squamous cell carcinoma in a dominant model in the Indian subcontinent, (**C**) Forest plot depicting the odds ratios (ORs), and 95% CI of the polymorphism, rs1048943/*CYP1A1* for its association with adenocarcinoma in a dominant model in the Indian subcontinent, and (**C’**) Forest plot depicting the odds ratios (ORs), and 95% CI of the polymorphism, rs1048943/*CYP1A1* for its association with squamous cell carcinoma in a dominant model in the Indian subcontinent. (**A**–**C**) are the representation of the analysis in the FE-model, while (**C’**) are in RE-model. The forest plots of the significant associations were given (*p* < 0.05*). The figures were generated in the ‘metafor’ package (http://www.metafor-project.org) of R software (https://cran.r-project.org/).

We found significant association of rs1048943 with lung Adenocarcinoma (AG + GG *vs.* AA: OR = 3.38, 95% CI = 1.63–6.25, *p* = 0.0001, Q^2^ = 1.99, *p*_*Het*_ = 0.16, I^2^ = 49.65%) (Table 3 and Fig. 2) and Lung Squamous carcinoma (AG + GG *vs.* AA: OR = 3.83, 95% CI = 2.15–6.82, *p* = 0.000005, Q^2^ = 2.72, *p*_*Het*_ = 0.07, I^2^ = 63.17%) in FE model (Table 3). Significant heterogeneity was observed for the variant. Therefore, meta-analysis fitted with the RE model showed a significant association of the variant with lung Squamous carcinoma (AG + GG *vs.* AA: OR = 3.91, 95% CI = 1.49–10.19, *p* = 0.005) only (Table 3 and Fig. 2).

**Table 3:** Results of the histological subtype-stratified meta-analysis of reported lung cancer variants in the Indian subcontinent.

			**Test for Association**				**Heterogeneity**		
**Variant ID/ Gene**	**Histological subtypes**	**Number**	**Genetic Model**	**Comparison**	**Model**	**OR (95% CI); *p-value***^**†**^	**I**^2^	**H**^2^	***p***_***het***_^**§**^
**del1/*GSTT1***	Squamous cell carcinoma	3	**Recessive**	**Null (-/-) vs Present (+ /-, + / +)**	Fixed	1.38 (0.7–2.74); *0.35*	17.69	1.21	*0.49*
	**Adenocarcinoma**	**3**			**Fixed**	**2.14 (1.04–4.41); *0.04***	0.79	1.01	*0.91*
	Small Cell Lung Carcinoma	2			Fixed	1.15 (0.44–3.03); *0.77*	26.08	1.36	*0.31*
del2/*GSTM1*	Squamous cell carcinoma	3	Recessive	Null (-/-) vs Present (+ /-, + / +)	Fixed	1.33 (0.74–2.4); *0.34*	0.11	1	*0.96*
	Adenocarcinoma	3			Fixed	1.32 (0.68–2.57); *0.41*	24.23	1.32	*0.48*
	Small Cell Lung Carcinoma	2			Fixed	1.53 (0.67–3.46); *0.31*	0.32	1	*0.77*
**rs4646903/*CYP1A1***	**Squamous cell carcinoma**	**4**	**Dominant**	**(TC + CC) vs TT**	**Fixed**	**1.82 (1.06–3.11); *0.03***	11.38	1.13	*0.71*
	Adenocarcinoma	4			Fixed	1.36 (0.68–2.7); *0.38*	20.93	1.26	*0.6*
	Small Cell Lung Carcinoma	2			Fixed	0.88 (0.34–2.29); *0.8*	0.001	1.02	*0.98*
**rs1048943/*CYP1A1***	**Squamous cell carcinoma**	**3**	**Dominant**	**(AG + GG) vs AA**	**Random**	**3.91 (1.49–10.19); *0.005***	63.17	2.72	***0.07***
	**Adenocarcinoma**	**3**			**Fixed**	**3.38 (1.83–6.25); *0.0001***	49.65	1.99	*0.16*

^†^p-value < 0.05*, 0.01**, 0.001***, OR, Crude Odds Ratio, 95% CI, 95% Confidence Interval, I^2^ and H^2^ are measures of heterogeneity. ^§^p_Het_ < 0.1 (Cochran’s Q test). Significant associations are depicted in bold.

#### Smoking status-stratified subgroup analysis:

**Figure 3:**
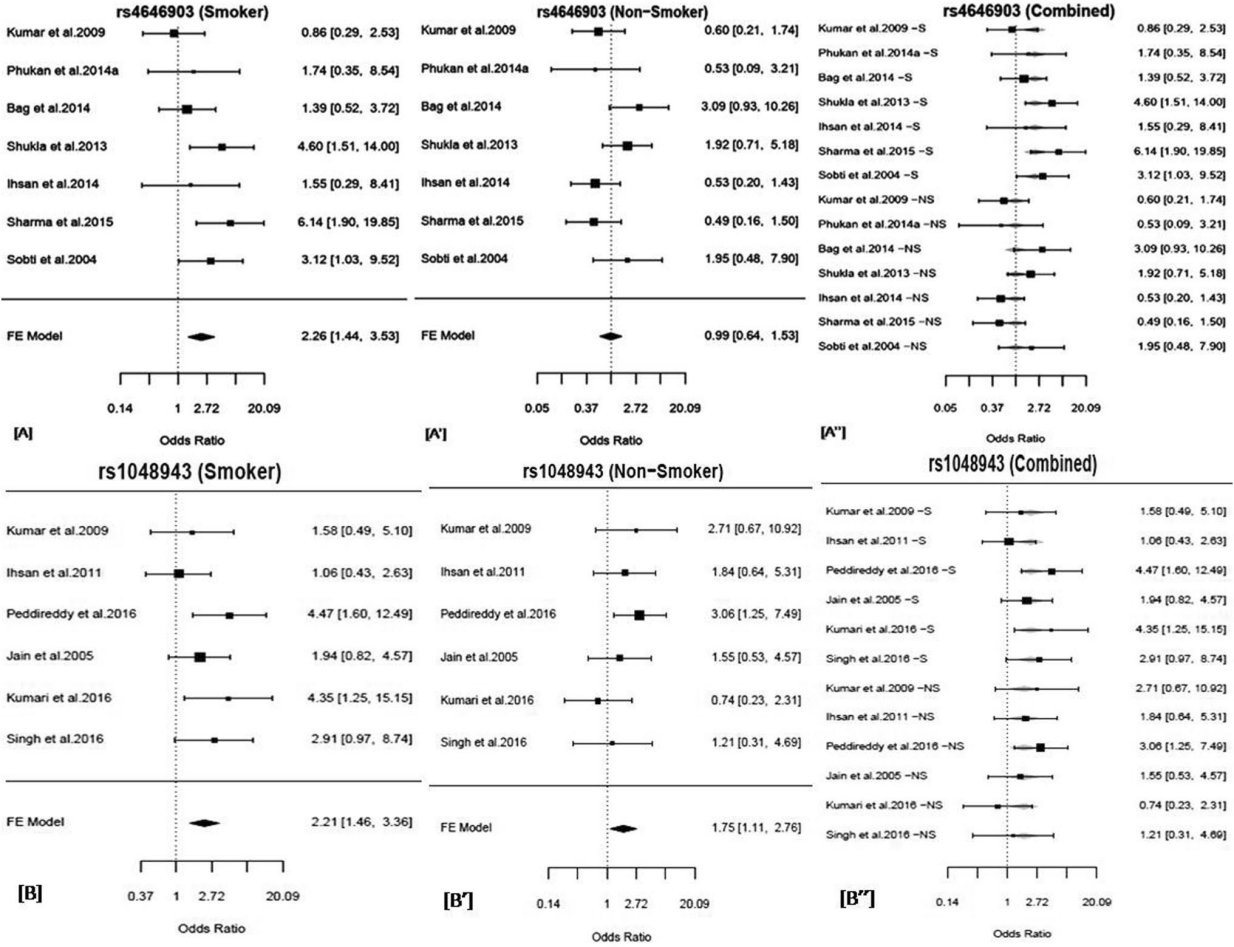
Forest plot depicting the odds ratios (ORs), and 95% CI of, rs4646903/*CYP1A1* for its association with lung cancer in a dominant model in the Indian subcontinent among (**A**) Smokers, (**A’**) Non-Smokers and (**A’’**) Combine forest plot of rs4646903/*CYP1A1* for its association with lung cancer stratified by smoking status in a dominant model in the Indian subcontinent, Forest plot depicting the odds ratios (ORs), and 95% CI of, rs1048943/*CYP1A1* for its association with lung cancer in a dominant model in the Indian subcontinent among (**B**) Smokers, (**B’**) Non-Smokers, and (**B’’**) Combine forest plot of rs1048943/*CYP1A1* for its association with lung cancer stratified by smoking status in a dominant model in the Indian subcontinent. The forest plots of the significant associations were given (*p* < 0.05*). The figures were generated in the ‘metafor’ package (http://www.metafor-project.org) of R software (https://cran.r-project.org/).

Using smoking status-stratified meta-analysis, we found a significant association of rs1048943 with lung cancer in both “Smoker” (AG + GG *vs.* AA: OR = 2.26, 95% CI = 1.44–3.53, *p* = 0.0004, Q^2^ = 1.74, *p*_*Het*_ = 0.17, I^2^ = 42.63%) and “Non-Smoker” (AG + GG *vs.* AA: OR = 1.75, 95% CI = 1.11–2.76, *p* = 0.02, Q^2^ = 1.35, *p*_*Het*_ = 0.49, I^2^ = 26.06%) subgroups (Table 4 and Fig. 3), with a stronger effect in smokers. Further, we did not find any significant effect modification by smoking (Table 5) by meta-regression analysis. Thus, the variant rs1048943 could be responsible for the metabolism of xenobiotics, present in both smokers and non-smokers that might be the reason for such confounding effect.

**Table 4:** The results of the subgroup analysis stratified by smoking status.

Variant ID/ Gene Symbol	Sub-groups	Number	Genetic Model	OR (95% CI); *p-value*^†^	I^2^	H^2^	*p*_*het*_^§^
del1/*GSTT1*	Smoker	9	Recessive	1.43 (0.93–2.22); *0.11*	31.58	1.46	*0.38*
	Non-Smoker			0.63 (0.39–1.04); *0.07*	43.89	1.78	*0.34*
del2/*GSTM1*	Smoker	9	Recessive	0.97 (0.65–1.44); *0.88*	4.49	1.05	*0.98*
	Non-Smoker			1.19 (0.77–1.82); *0.43*	3.48	1.04	*0.98*
**rs4646903/*CYP1A1***	**Smoker**	7	Dominant	**2.26 (1.44–3.53); *0.0004***	42.63	1.74	*0.17*
	Non-Smoker			0.99 (0.64–1.53); *0.95*	50.39	2.02	*0.11*
**rs1048943/*CYP1A1***	**Smoker**	6	Dominant	**2.21 (1.46–3.36); *0.002***	37.39	1.59	*0.29*
	**Non-Smoker**			**1.75 (1.11–2.76); *0.02***	26.06	1.35	*0.49*

^†^p-value < 0.05*, 0.01**, 0.001***, OR, Crude Odds Ratio, 95% CI, 95% Confidence Interval, I^2^ and H^2^ are.

measures of heterogeneity. ^§^p_Het_ < 0.1 (Cochran’s Q test). Significant associations are depicted in bold.

**Table 5:** The results of the effect modification of variants on lung cancer by smoking status.

Moderator variable	Genetic Model	Number	Moderator Effect Size (θ)	θ low	θ high	*p-value*^†^
**del1/*GSTT1***	**Recessive**	**18**	**0.82**	**0.16**	**1.48**	***0.015***
del2/*GSTM1*	Recessive	18	− 0.21	− 0.79	0.38	*0.49*
**rs4646903/*CYP1A1***	**Dominant**	**14**	**0.83**	**0.2**	**1.45**	***0.01***
rs1048943/*CYP1A1*	Dominant	12	0.23	− 0.38	0.85	*0.46*

^†^p-value < 0.05*, 0.01**, 0.001***, OR, Crude Odds Ratio, 95% CI, 95% Confidence Interval, I^2^ and H^2^ are measures of heterogeneity. ^§^p_Het_ < 0.1 (Cochran’s Q test). Significant associations are depicted in bold.

#### *CYP1A1*/rs4646903 (dominant model)

We found a significant association of the variant with overall lung cancer risk without any significant heterogeneity (TC + CC vs TT: OR = 1.48, 95% CI = 1.93–1.95, *p* = 0.005, *p*_*FDR*_ = 0.07, Q^2^ = 1.19, *p*_*Het*_ = 0.84, I^2^ = 15.98% (Table 2 and Fig. 1). The variant also showed significant association with lung cancer after multiple testing adjustment by Benjamini–Hochberg FDR correction (*p*_*FDR*_ = 0.07). We did not find any significant publication bias from the inspection of the funnel plots (Fig. 1) and Egger’s Test (*p* = *0.35*).

#### Association with lung cancer histological subtypes

Our analysis found a significant association of the variant with lung Squamous carcinoma only without heterogeneity (TC + CC vs TT: OR = 1.82, 95% CI = 1.06–3.11, *p* = 0.03, Q^2^ = 1.13, *p*_*Het*_ = 0.71, I^2^ = 11.38%) only (Table 3 and Fig. 2).

#### Smoking status-stratified subgroup analysis

The variant showed significant association with lung cancer in smoker subgroup (TC + CC vs TT: OR = 2.26, 95% CI = 1.44–3.53, *p* = 0.0004, Q^2^ = 1.74, *p*_*Het*_ = 0.17, I^2^ = 42.63%) only (Table 4 and Fig. 3). The meta-regression analysis showed significant effect modification by smoking (*p* = 0.01) (Table 5).

#### *GSTT1*/del1 (recessive model)

Meta-analysis fitted with FE model showed a nominal association of the variant with overall lung cancer [Null (−/−) vs Present [(+ /−) + (+ / +)]: OR = 1.36, 95% CI = 1.03–1.79, *p* = 0.028, *p*_*FDR*_ = 0.42, Q^2^ = 1.45, *p*_*Het*_ = 0.49, I^2^ = 31.1%) without any evidence of heterogeneity (Table 2 and Supplementary Information, Fig. S4). We found no significant publication bias from the inspection of the funnel plots (Supplementary Information, Fig. S4) and Egger’s Test (*p* = 0.93).

#### Association with lung cancer histological subtypes

Meta-analysis stratified by histological subtypes showed an association of the variant with lung Adenocarcinoma (Null (−/−) vs Present [(+ /-) + (+ / +)]: OR = 2.14, 95% CI = 1.04–4.41, *p* = 0.04, Q^2^ = 1.01, *p*_*Het*_ = 0.91, I^2^ = 0.79%) only (Table 3 and Fig. 2).

#### Smoking status-stratified subgroup analysis

We found no significant association of the variant with the smoking status-stratified subgroups, i.e. Smokers and Non-Smokers (Table 4 and Fig. 3). However, it showed significant effect modification by smoking in a meta-regression analysis (*p* = 0.015) (Table 5).

#### GSTM1/del2 (recessive model)

Using FE meta-analysis, we found a nominal association of the variant with overall lung cancer (Null (-/-) vs Present [(+ /-) + (+ / +)]: OR = 1.38, 95% CI = 1.09–1.75, *p* = 0.008, *p*_*FDR*_ = 0.42, Q^2^ = 1.88, *p*_*Het*_ = 0.29, I^2^ = 46.9%) without significant heterogeneity (Table 2 and Supplementary Information, Fig. S5). In our analysis, Bhardwaj et al. 2018 seems to be an outlier but did not cause significant heterogeneity as the study's sample size was small. We did not find any significant publication bias from the inspection of the funnel plots (Supplementary Information, Fig. S5) and Egger’s Test (*p* = 0.93).

#### Association with lung cancer histological subtypes

We found no association of the variant with any of the histological subtypes included in the study (Table 3).

#### Smoking status-stratified subgroup analysis

We found no significant association of the variant with any smoking status-stratified subgroups, i.e. Smokers and Non-Smokers (Table 4). The meta-regression analysis showed no significant effect modification by smoking (Table 5).

#### GSTP1/rs1695 (dominant model)

We found a marginal association of the variant with overall lung cancer risk (GG + AG vs AA: OR = 1.84, 95% CI = 1.07–3.16, *p* = 0.03, *p*_*FDR*_ = 0.15, Q^2^ = 5.02, *p*_*Het*_ = 0.007, I^2^ = 80.08%), using FE meta-analysis (Table 2). We observed significant heterogeneity and performed RE meta-analysis, which showed a lack of association of rs1695 with lung cancer (Table 2 and Supplementary Information, Fig. S6). Due to an insufficient number of studies, the assessment of publication bias was not reliable. Subgroup analysis was also not done due to the lack of sufficient data.

#### DKK2/rs17037102 (additive and dominant models)

Similarly, in FE meta-analysis, we found a marginal association of rs17037102 with overall lung cancer risk without any significant heterogeneity, in both the additive (AA vs AG vs GG : OR = 1.81, 95% CI = 1.03–3.15, *p* = 0.04, *p*_*FDR*_ = 0.53, Q^2^ = 1.00, *p*_*Het*_ = 0.99, I^2^ = 0.00002%) and the dominant (AA vs AG + GG: OR = 1.82, 95% CI = 1.03–3.22, *p* = 0.04, *p*_*FDR*_ = 0.16, Q^2^ = 1.00, *p*_*Het*_ = 0.99, I^2^ = 0.004%) models (Table 2 and Supplementary Information, Fig. S7). Subgroup analysis and assessment of publication bias was not done due to lack of sufficient data.

#### Summary of the significant findings

- *GSTT1* (del1)—associated with overall lung cancer risk, Adenocarcinoma, and showed significant effect modification by smoking status.
- *CYP1A1* (rs4646903)—associated with overall lung cancer risk, Squamous cell carcinoma, lung cancer in Smokers, and showed significant effect modification by smoking status.
- *CYP1A1* (rs1048943)—associated with overall lung cancer risk, both Adenocarcinoma and Squamous cell carcinoma, and in smoker and non-smoker subgroups.
- *GSTM1* (del2), *GSTP1* (rs1695), and *DKK2* (rs17037102)—associated with overall lung cancer risk.

### Association of rs1048943/*CYP1A1* with lung cancer in a case–control dataset of East Indian population

The study sample's detailed demographic and clinical attributes from East India are summarised (Supplementary Information, Table S2). Out of the 2 variants confirmed to be associated by the meta-analysis after FDR correction, namely rs4646903/*CYP1A1* and rs1048943/*CYP1A1,* the latter showed significant heterogeneity (Q = 1.93, I^2^ = 48.32, *p* = 0.092). We hypothesised that this heterogeneity might be explained by looking at covariate-specific and subgroup-stratified analysis. One of the major reasons for heterogeneity in the crude analysis is the uneven distribution of confounder/subgroups across studies. Hence, to understand the source of heterogeneity, we genotyped this polymorphic variant in a case–control dataset among smokers comprising 101 lung cancer cases and 413 healthy controls from a representative East Indian sample population. Several relevant covariates such as age, sex, pack-years of smoking were measured for their effect on lung cancer risk.

#### Genotyping and quality control

We genotyped rs1048943 in our sample set. The representative image of the RFLP analysis is depicted in (Supplementary Information, Fig. S8). To determine the quality of our genotyping, we assessed HWE in controls and found it to be consistent with HWE (*p* > 0.05). We found no significant association of the variant with lung cancer among smokers (GG + GA *vs* AA: OR = 1.33, 95% CI = 0.825–2.16; *p*_*adj*_ = 0.24) adjusted for age, ethnicity, smoking intensity in pack-years, alcohol consumption, tobacco and betel quid chewing, and asbestos exposure in the dominant model (Supplementary Information, Table S3).

#### Covariate-stratified subgroup analysis

Using covariate-stratified subgroup analysis, we found no significant association of rs1048943 in any of the covariate subgroups (Supplementary Information, Table S4).

#### Association with lung cancer histological subtypes

We found a nominal association of rs1048943/*CYP1A1* with lung Adenocarcinoma (ADC) among smokers in the dominant (GG + GA *vs* AA: OR = 1.99; 95% CI = 1.10–3.63; *p* = 0.024) effect model (Supplementary Information, Table S5). We have performed an age-adjusted analysis restricted in males only, which showed a significant association of rs1048943 (*CYP1A1*) with lung Adenocarcinoma (OR = 2.97, 95% CI = 1.35–6.69, *p* = 0.007) among smokers (Supplementary Information, Table S5). Further, adjusting for age, pack-years of smoking and ethnicity, we also found a significant association of rs1048943 with Adenocarcinoma (OR = 2.08, 95% CI = 1.08–4.02, *p* = 0.03) in smokers. Interestingly, we found a negative effect of age (β = -0.14) on Adenocarcinoma in smokers, which signifies rs1048943 (CYP1A1) to confer risk of developing lung adenocarcinoma in young male smokers. Thus, the results indicate a potential role of rs1048943/*CYP1A1* in a specific histological subtype of lung cancer, indicating the disease's genetic heterogeneity and variability.

### Meta-analysis of rs1048943 (CYP1A1) in world population

A literature search with the specific keywords revealed a total of 2617 hits for the variant rs1048943 (*CYP1A1*) published till 31^st^ December 2019, worldwide. Our case–control association was included in the pool of hits, which increased the total number of hits for rs1048943 (*CYP1A1*) to 2618. Following the specific inclusion/exclusion criteria, 40 studies with 10,458 cases and 10,871 controls were selected for the meta-analysis (Supplementary Information Fig. S9). All the covariate and demographic data for rs1048943 are listed in a tabular form (Supplementary Information, Table S6).

In the FE meta-analysis, we found a nominal association of rs1048943 with overall lung cancer risk (AG + GG *vs* AA: OR = 1.21, 95% CI = 1.04–1.41, *p* = 0.01, Q^2^ = 1.74, *p*_*Het*_ = 0.08, I^2^ = 42.67%) (Supplementary Information, Table S7 and Fig. S10). Since there was evidence for heterogeneity, we did a RE meta-analysis and found no association of rs1048943 (AG + GG *vs* AA: OR = 1.20, 95% CI = 0.98–1.47, *p* = 0.07) with overall lung cancer risk (Supplementary Information, Table S7 and Fig. S10). The RE model is known to have lower power, which could potentially explain the lack of significant association of rs1048943/*CYP1A1* with overall lung cancer. No significant publication bias was observed from the inspection of the funnel plots (Supplementary Information, Fig. S10) and Egger’s test (*p* = 0.23). Further, we stratified the crude genotype counts of rs1048943/*CYP1A1* by the selected studies' geographical region/country. We found a significant association (*p* < 0.05) of the variant with lung cancer in the Indian and Australian population (Supplementary Information, Table S8 and Fig. S11).

To study the association of the variant in more homogeneous strata, we performed histology and smoking status–stratified subgroup analysis as given below.

#### Association with lung cancer histological subtypes:

A significant association of rs1048943 with lung Adenocarcinoma (AG + GG *vs* AA: OR = 1.35, 95% CI = 1.03–1.77, *p* = 0.028, Q^2^ = 1.98, *p*_*Het*_ = 0.032, I^2^ = 49.55%) and Lung Squamous carcinoma (AG + GG *vs* AA: OR = 1.50, 95% CI = 1.14–1.99, *p* = 0.004, Q^2^ = 2.05, *p*_*Het*_ = 0.02, I^2^ = 51.33%) were found in FE model. The RE meta-analysis showed a significant association with only lung Squamous carcinoma; (AG + GG *vs* AA: OR = 1.53, 95% CI = 1.02–2.30, *p* = 0.04) only (Supplementary Information, Table S9 and Fig. S12).

#### Smoking status-stratified subgroup analysis:

A significant association of rs1048943 in “Smoker” (AG + GG *vs* AA OR = 1.57, 95% CI = 1.16–2.11, *p* = 0.003, Q^2^ = 2.33, *p*_*Het*_ = 0.22, I^2^ = 57.14%) subgroup was observed and “Non-Smoker” (AG + GG *vs* AA: OR = 1.39, 95% CI = 0.99–1.93, *p* = 0.051, Q^2^ = 1.41, *p*_*Het*_ = 0.42, I^2^ = 29.54%) subgroups were found (Supplementary Information, Table S10 and Fig. S13) with no effect modification by smoking (*p* = 0.59) (Supplementary Information, Table S11).

## Discussion

Our study presents the first comprehensive meta-analysis of 18 variants of 11 genes across 39 studies from the Indian subcontinent that provides an insight into the combined effect of each variant on overall and covariate-stratified lung cancer risk in the region. The lack of significant publication bias confirms that the results were not overestimated under the influence of any bias in the published articles. Although *GWAS* data mining revealed no significant association of rs1048943/*CYP1A1* with lung cancer, it showed a significant association of the *CYP1A1* gene with hypertension and habitual coffee consumption. Therefore, the variant's association with lung cancer could be modified by coffee consumption or smoking tobacco. The variant rs1048943/*CYP1A1* was associated with lung cancer risk in East Asians^51^, which shows the colinearity of this study's findings to the present study as discussed here. The *CYP1A1* (Cytochrome P450, family 1, member A1; 15q22-24) gene encodes a bulky phase I endoplasmic xenobiotic metabolism enzyme present in lung tissue. The enzyme catalyses the activation of reactive electrophilic compounds, including benzo[a]pyrenes and PAHs present in tobacco smoke ^52^. It promotes DNA adducts formation, which imparts a genotoxic effect that could lead to DNA lesions and cause lung cancer. The variant rs1048943A > G of *CYP1A1* locus causes a single amino acid substitution (*Ile* > *Val*) in the heme-binding region, which increases enzyme activity, enhancing the activation of procarcinogens in tobacco smoke. It influences the metabolism of environmental carcinogens, such as tobacco smoke, that modifies lung cancer susceptibility^52^.

The superfamily of glutathione-S-transferases (GSTs) comprises multifunctional enzymes that catalyse the conjugation of reduced tripeptide glutathione to various electrophilic and hydrophobic substrates resulting in their detoxification and effective elimination from the cell. Thus, they help to reduce the carcinogenic load accumulated due to smoking from the cells. The null genotype of the deletion polymorphisms of glutathione-S-transferase theta 1 (*GSTT1*) and glutathione-S-transferase mu 1 (*GSTM1*) is frequently associated with lung cancer with evidence of effect modification by tobacco smoking. The null genotype is responsible for the lack of the enzyme within the cell, conferring a higher risk of lung cancer. Inconsistent reports on the association of *GSTT1* (del1) and *GSTM1* (del2) null genotypes led to confusion regarding their correct effect on the disease pathogenesis^53,54^. Ethnicity/racial differences in the association of *GSTT1* null genotype with lung cancer has been reported where the frequency of the null genotype was significantly higher in Asians than in Caucasians^55^.

The gene Dickkopf-related protein 2 (*DKK2*) encodes a secretory protein belonging to the Dickkopf family. The protein DKK2 bears two cysteine-rich regions and is involved in embryonic development through the Wnt/β-catenin signalling pathway. DKK2 exhibits a bimodal function as an agonist or antagonist of the Wnt/β-catenin signalling pathway^56^ depending on the cellular context and the presence of the co-factor kremen2. The activity of DKK2 is modulated by the Wnt co-receptor, LDL-receptor related protein 5 (LRP5) and -6 (LRP6)^57^. Aberrant expression of DKK2 has been observed in many tumours, including epigenetic silencing of the expression of DKK2 in ovarian carcinoma^58^, hepatocellular carcinoma^59^, and renal carcinoma^60^. RNAi-mediated silencing of *DKK2* is frequently observed in tongue squamous cell carcinoma^61^ and oesophageal adenocarcinoma^62^. These reports are suggestive of the anti-tumour effect of DKK2. However, the upregulation of DKK2 promotes cell proliferation and invasion through the Wnt signalling pathway in prostate cancer^63^, Ewing sarcoma^64^, and colorectal cancer^65^. Thus, the cellular context-dependent function of DKK2 is very complex, which is evident from the above examples. DKK2 has been found to promote angiogenesis, distinct from VEGF-dependent angiogenesis^66^, forming closer interconnections of the vessels.

Interestingly, Dkk2-induced blood vessels consistently show higher coverage of endothelial cells (ECs) by pericytes and smooth muscle cells (SMCs), which are involved in vessel maturity and stability. Dkk2-mediated angiogenesis consists of a signalling cascade induced through LRP6-mediated APC/Asef2/Cdc42 activation. DKK2 promotes tumour progression by suppressing cytotoxic immune cell activation in colorectal carcinoma^67^ and NSCLC^68^ with *APC* mutations. In a recent study^26^, the heterozygous genotype of rs17037102/*DKK2* and rs419558/*DKK2* confer an increased risk of lung cancer. A combination of all the 3 genotypic variants of DKK2 confers a four-fold increase in lung cancer risk.

The protein encoded by *XRCC1* (X-ray repair cross-complementing 1; 19q13.31) performs an efficient repair of single-strand DNA breaks formed by the exposure to ionising radiation and alkylating agents. XRCC1 interacts with DNA ligase III, polymerase beta and poly (ADP-ribose) polymerase to participate in the base excision repair. The protein plays a role in DNA processing during meiosis and DNA recombination in germinal epithelial cells. Moreover, *XRCC1* harbours a rare microsatellite polymorphism, which is associated with varying radiosensitivity in cancer^69^. Polymorphisms of *XRCC1*, like Arg194Trp (exon 7), Arg280His (exon 10) and Arg399Gln (exon 11), were reported to confer increased risk to lung cancer^70–72^ with inconsistencies across different populations^73–77^.

The association of variants with different histological subtypes of lung cancer revealed del1/*GSTT1* to be associated with lung adenocarcinoma, rs4646903/*CYP1A1* with lung squamous cell carcinoma while rs1048943/*CYP1A1* with both lung adenocarcinoma and lung squamous cell carcinoma. Thus, stratification of the genotypes based on the histological subtypes of lung cancer adjusted for age, pack-years of smoking and ethnicity has improved risk assessment potential. Identification of subtypes specific genetic risk markers helps to design targeted early detection and prevention strategies. Moreover, identifying histotype-associated genetic markers may define the mechanism underlying the currently unknown origins of morphological variations that could develop personalised treatment modalities for subtype-specific lung cancer cases.

Furthermore, subgroup analysis of 4 variants stratified by smoking status revealed rs1048943 of the *CYP1A1* gene to be significantly associated with lung cancer in smokers and non-smokers. However, the meta-regression analysis revealed the absence of any effect modification of rs1048943 on lung cancer by smoking, implying that the polymorphism has no modifier effect on lung cancer. The variant rs4646903 of the *CYP1A1* gene show an association with lung cancer in smokers only. Interestingly, significant effect modification of del1 of the *GSTT1* gene and rs4646903 of the *CYP1A1* gene on lung cancer by smoking was observed by meta-regression analysis, which suggested the importance of the variants in modifying the risk of lung cancer by smoking status.

Based on the meta-regression analysis, there is no significant effect modification for the remaining variants, although it can be surmised that there may be interaction in the biological mechanisms leading to lung cancer. The variant rs1048943 could be involved in the metabolism of xenobiotics present in both smokers and non-smokers, which might be the reason for such confounding effects. Hence, we believe that the variant has biological relevance in lung carcinogenesis, but more extensive analysis of different covariates, including smoking in larger samples, are required to dissect its actual effect on lung carcinogenesis. Subgroup analysis based on covariates, such as age, sex, ethnicity, exposure types and dose, was not done due to lack of sufficient reports on the population of the Indian subcontinent. The Indian subcontinent consists of a highly heterogeneous population with considerable admixture among different ethnicities, which could modify the population's linkage disequilibrium structure^78^. This could contribute to significant heterogeneity between the studies.

The variant rs1048943A > G of *CYP1A1* locus is a non-synonymous polymorphic variant, which imparts an individual effect on lung cancer risk in various populations^31,79,80^. A case–control analysis followed the meta-analysis in the East Indian sample population among smokers, which revealed no association of rs1048943A > G of *CYP1A1* with overall lung cancer risk among smokers. However, the variant rs1048943 showed a significant association with lung adenocarcinoma in smokers adjusted for various covariate factors. Thus, our case–control study reveals rs1048943/*CYP1A1* as a histological subtype-specific variant for lung cancer in the East Indian population, potentially targeting personalised therapy and histology-specific drug designing for lung cancer patients. The finding shows colinearity with the outcomes of the current meta-analysis. The studies included in this meta-analysis, reported from the Eastern region of the subcontinent, also shows a lack of association of the risk genotype (GG) of the polymorphic variant rs1048943/*CYP1A1* as summarised in (Table 1 and Supplementary Information, Table S1)^81,82^. Our replication meta-analysis across the world population justifies the role of the variant rs1048943 (CYP1A1) in conferring lung cancer risk among smokers with a higher power. Interestingly, rs1048943 (*CYP1A1*) shows no effect modification by smoking status on lung cancer risk that is indicative of the association in smokers as a random occurrence by chance, or it might be involved in the metabolism of other xenobiotics in both smokers and non-smokers, leading to this confounding effect of smoking.

In the larger sample size, the variant rs1048943 (*CYP1A1*) shows an association with squamous cell carcinoma, which is indicative of a population-specific effect of the variant on different histological subtypes of lung cancer. The association of rs1048943 (*CYP1A1*) across various populations identifies the relevance of the variant in lung cancer risk in a population-specific manner, which could be critical in designing personalised treatment and precision medicine for patients of diverse populations.

Interestingly, out of 11 selected genes for meta-analysis, 5 belong to the xenobiotic metabolism pathway, 3 belong to the DNA repair pathway, and 3 belong to the Wnt/β-catenin pathway regulating various physiological aspects of lung cancer. The xenobiotic metabolism and DNA repair pathways could be the significant ‘modifier’ and ‘driver’ pathways leading to altered gene-environment interaction and lung cancer development. Genes of xenobiotic metabolism pathways are involved in the metabolism and detoxification of tobacco smoke components to reduce intracellular carcinogenic load. Some genes of the xenobiotic metabolism pathway also induce bio-activation of procarcinogens into potent carcinogens that can quickly form DNA adducts and subsequent mutagenesis. The genes belonging to the DNA repair pathway repairs DNA damage induced by tobacco smoking and radiation. Detailed text mining of the available reports following the inclusion criteria revealed the association of xenobiotic metabolism genes (XMG) and DNA repair genes (DRG) to the risk of lung cancer development.

In the current study, we could not perform subgroup and meta-regression analysis for other covariate risk factors for all the variants due to insufficient data in the selected studies. Moreover, we could not adjust for any of the covariates in the meta-analysis due to insufficient data. The subtype-specific polymorphic variant identification obtained in the current meta-analysis would suffice personalised therapy and precision medicine development. Identifying genetic variants for which there is evidence of influence on lung cancer risk through meta-analysis may provide new insights into the fundamental biological pathways involved in developing lung cancer to help future research. Further, identifying lung cancer risk variants may also help assess risk scores for accurate population risk stratification and decision-making, which could be of potential value in targeting primary prevention and lung cancer screening modalities in a population-specific manner.

## Supplementary Information

Supplementary Information.

## Data Availability

All data generated or analysed during this study are included in this article and its supplementary information files. The additional raw input data files will be available from the corresponding authors on request.

## Code availability

The R scripts/codes used for the analysis will be available from the corresponding author on request.
