# Supplementary material for: A comprehensive meta-analysis and a case–control study give insights into genetic susceptibility of lung cancer and subgroups
Source: Sci Rep. 2021 Jul 16;11:14572. doi: 10.1038/s41598-021-92275-z (PMC8285487; doi:10.1038/s41598-021-92275-z)
Supplement: Supplementary file 1 — Supplementary Information. [file 41598_2021_92275_MOESM1_ESM.pdf]

A comprehensive meta-analysis and a case-control study give insights into genetic susceptibility of lung cancer and subgroups

Supplementary Information

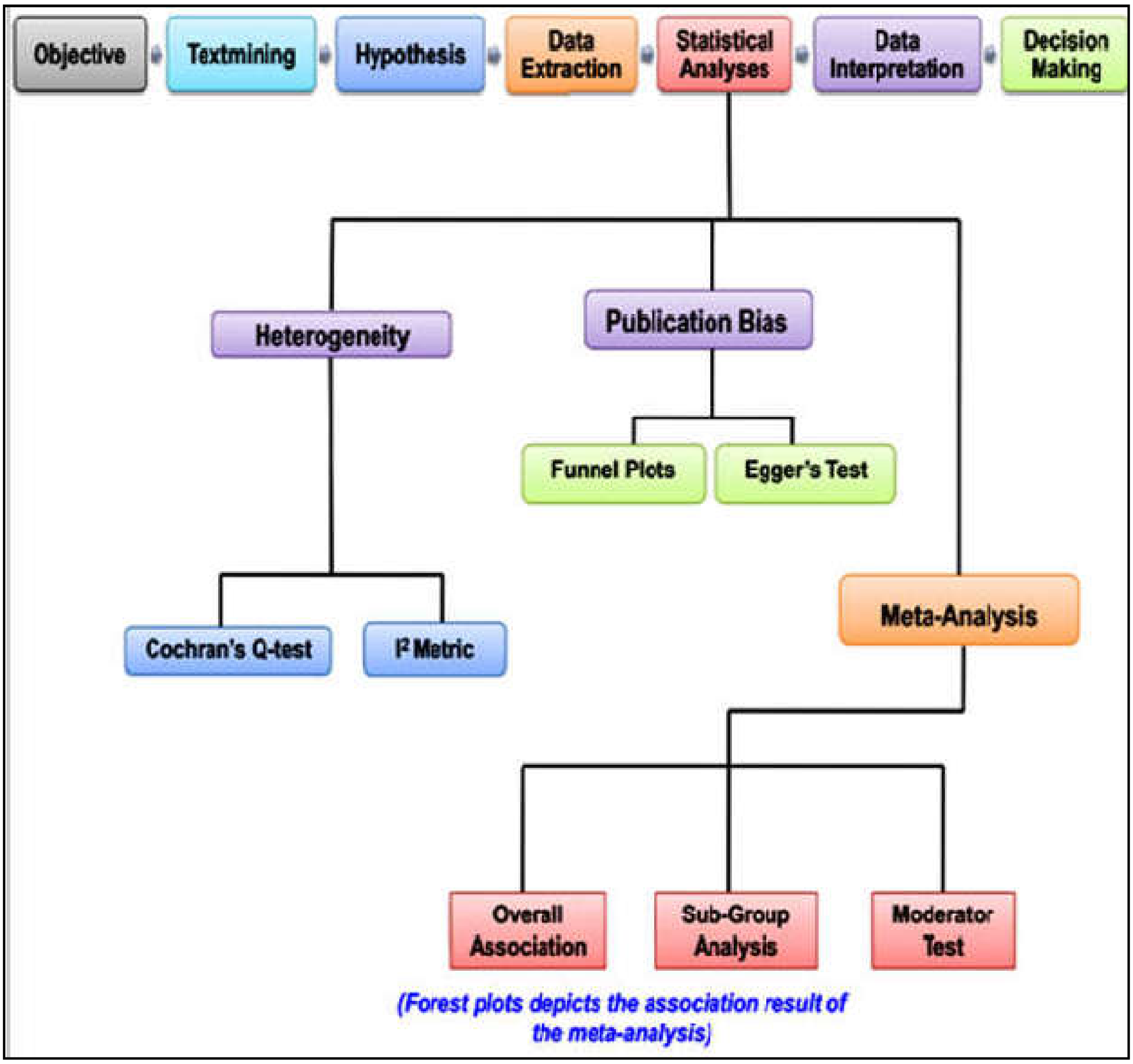

Fig. S1 The pipeline of meta-analysis.

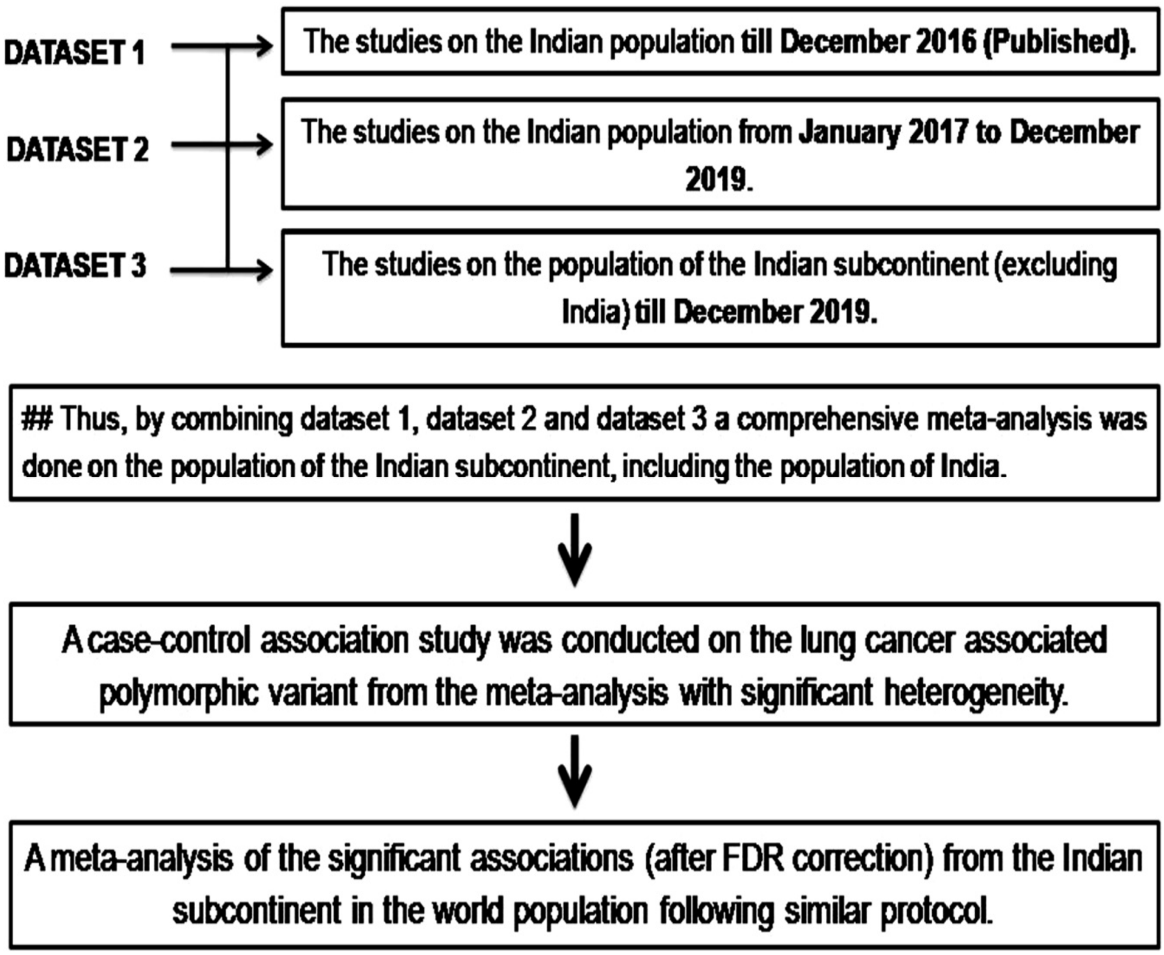

Fig. S2 The workflow of analysis explaining the comprehensive meta-analysis presented in this section. The data to conduct the comprehensive meta-analysis includes dataset 1 and dataset 2, as mentioned above.

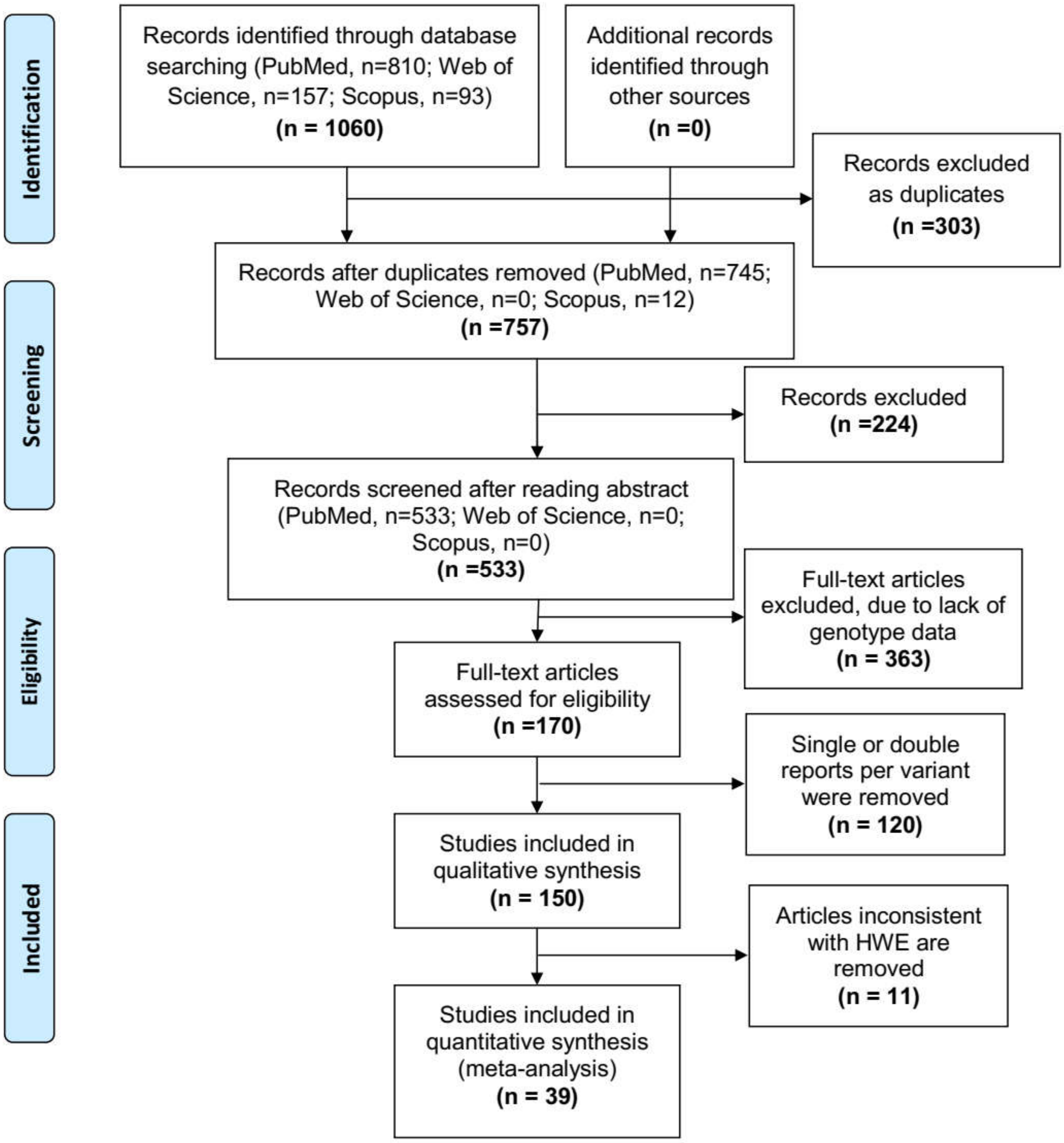

Fig. S3 The pathway of selecting studies for meta-analysis on candidate association studies reported from the Indian subcontinent. The flow diagram is constructed as per the PRISMA guidelines [**Source:** Moher, D., Liberati, A., Tetzlaff, J., Altman, D. G. & Group, P. Preferred reporting items for systematic reviews and meta-analyses: the PRISMA statement. *BMJ* **339**, b2535, doi:10.1136/bmj.b2535 (2009)].

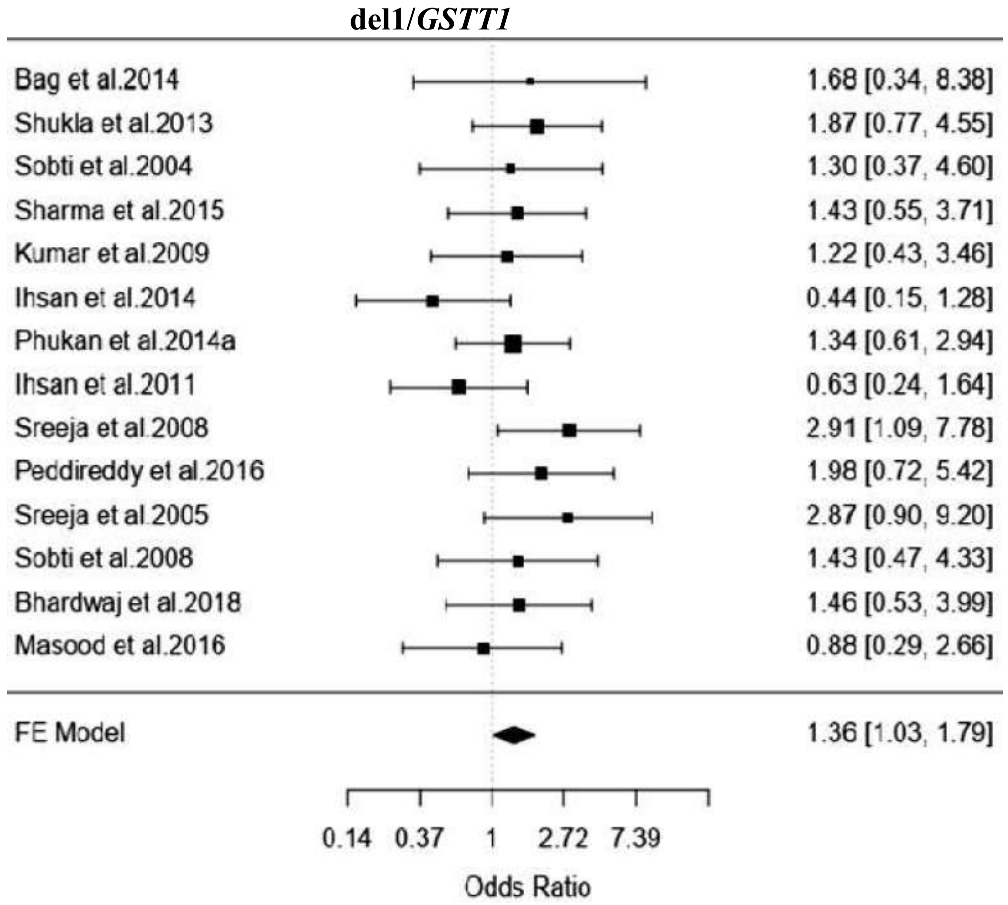

[A].

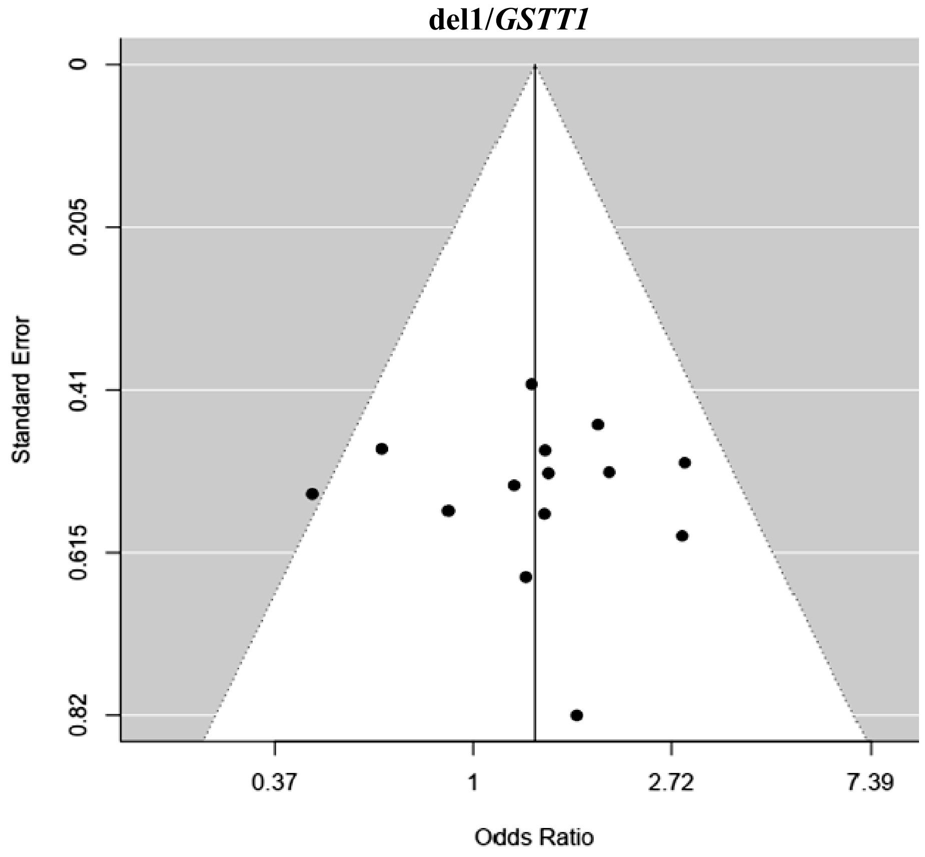

[A'].

Fig. S4 Forest plot depicting the odds ratios (ORs), and 95% CI of the polymorphism, **del1/*GSTT1*** for its association with overall lung cancer risk in the Indian subcontinent **[A] in a fixed-effect model**, **[A'] Funnel plot** that shows no evidence of publication bias between the studies reporting the polymorphism **del1/*GSTT1***. **The forest plots of the significant associations (*p* < 0.05) are given.** The figures were generated in the 'metafor' package (http://www.metafor-project.org) of R software (https://cran.r-project.org/)

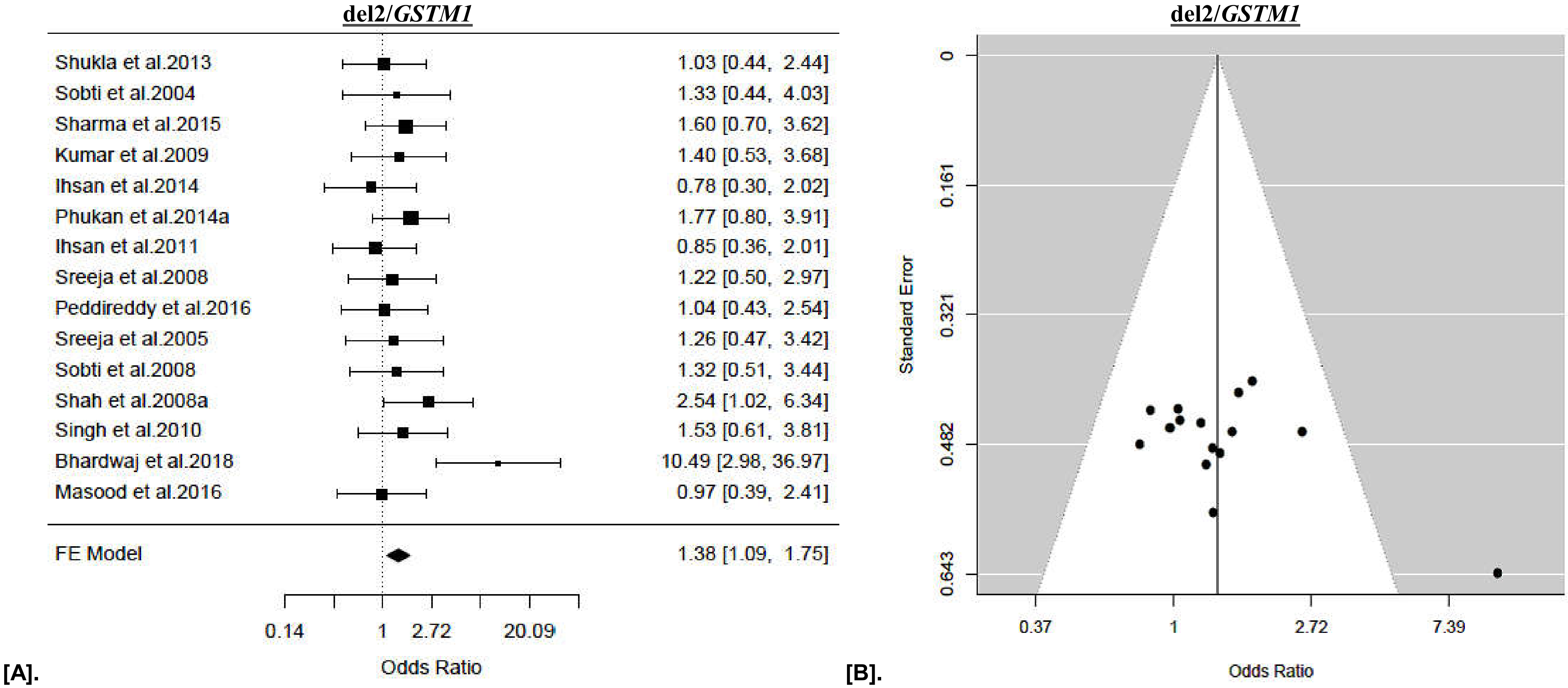

Fig. S5 Forest plots depicting the odds ratios (ORs), and 95% CI of the deletion polymorphism **del2/*GSTM1*** for its association with overall lung cancer risk in the Indian subcontinent, **[A] in a fixed-effect model [B] Funnel plot** that shows no evidence of publication bias between the studies reporting the polymorphism **del2/*GSTM1***. The results are obtained in a recessive genetic model. **The forest plots of the significant associations (*p* < 0.05) are given.** The figures were generated in the 'metafor' package (http://www.metafor-project.org) of R software (https://cran.r-project.org/)

rs1695/*GSTP1*

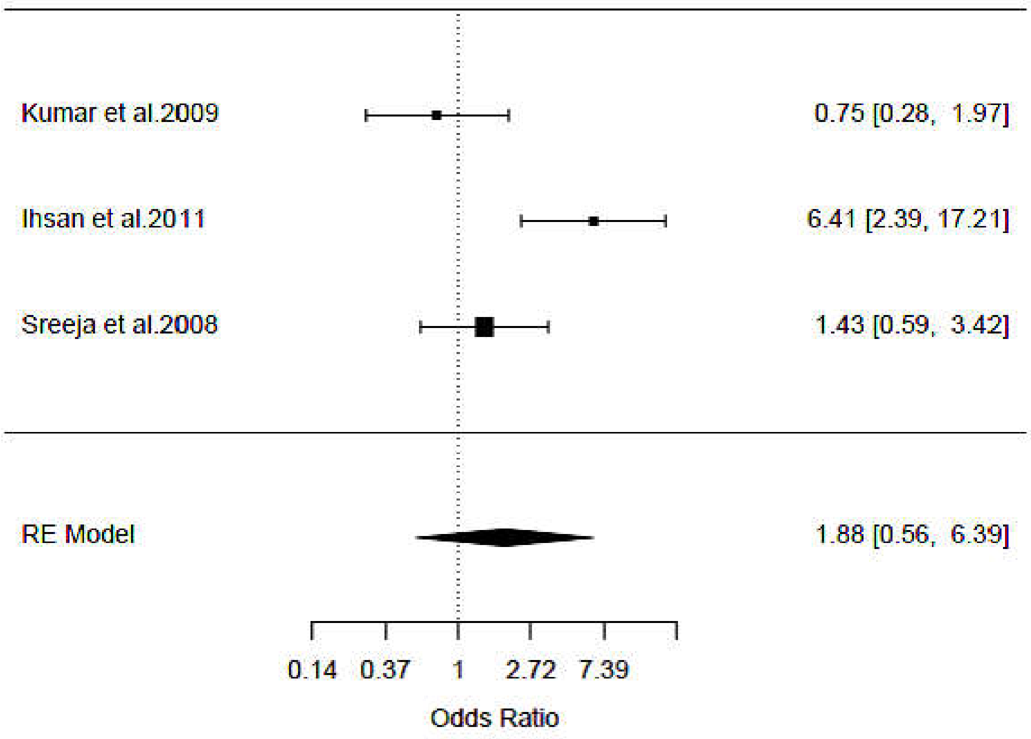

[A].

Fig. S6 Forest plots depicting the odds ratios (ORs), and 95% CI of the deletion polymorphism **rs1695/*GSTP1*** for its association with overall lung cancer risk in the Indian subcontinent, **[A] in a random-effects model**. The results are obtained in a dominant genetic model. **The forest plots of the significant associations (*p* < 0.05) are given.** The figures were generated in the 'metafor' package (http://www.metafor-project.org) of R software (https://cran.r-project.org/)

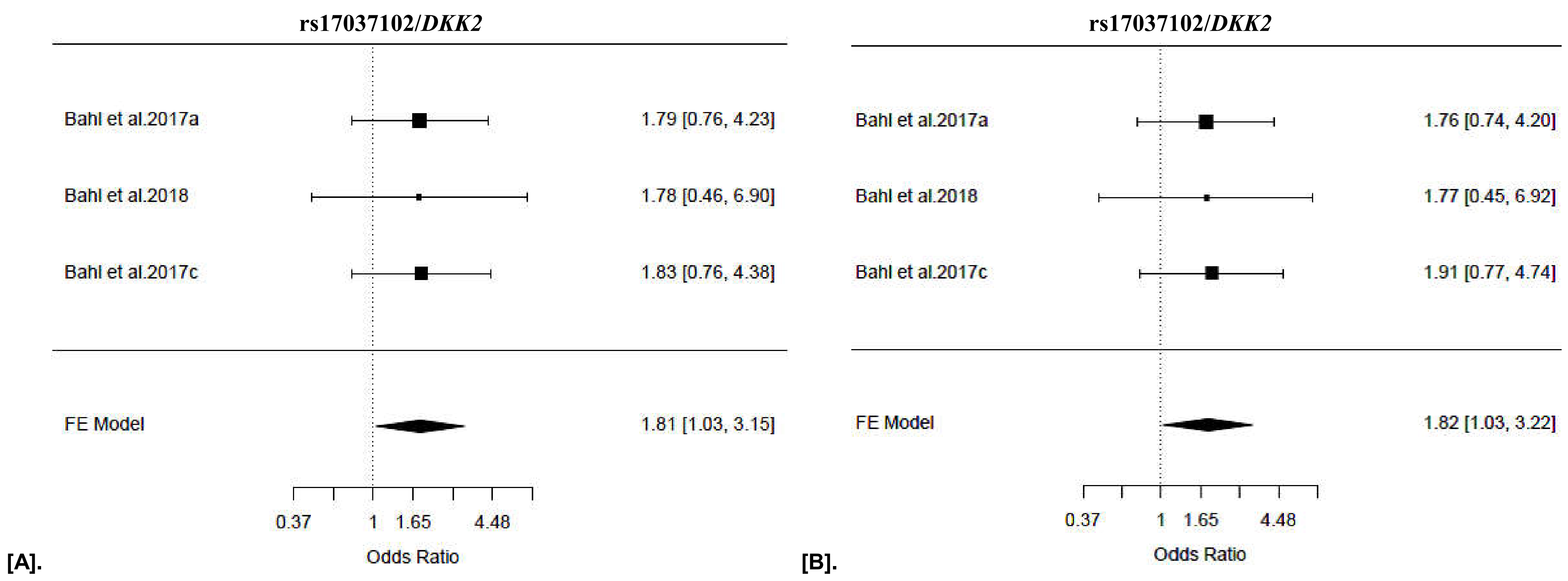

Fig. S7 Forest plots depicting the odds ratios (ORs) and 95% CI of the polymorphism, **rs17037102/ *DKK2*** for its association with overall lung cancer risk in the Indian subcontinent, **[A] in an additive genetic model, [B] in a dominant genetic model.** *The results are generated in fixed-effect model meta-analysis*. **The forest plots of the significant associations (p < 0.05) are given.** The figures were generated in the 'metafor' package (http://www.metafor-project.org) of R software (https://cran.r-project.org/)

1048943/*CYP1A1*

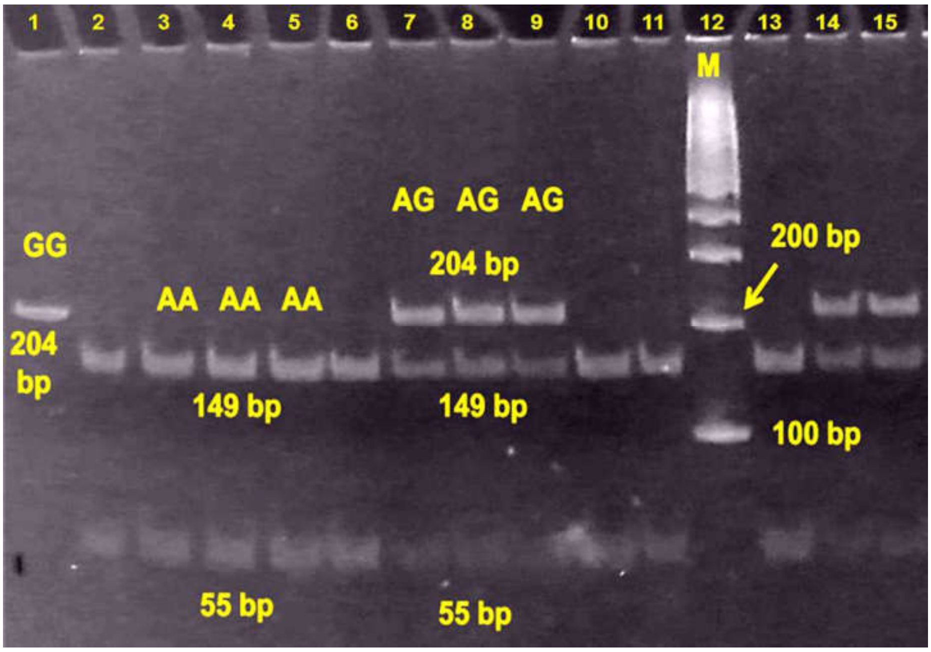

Unprocessed Image

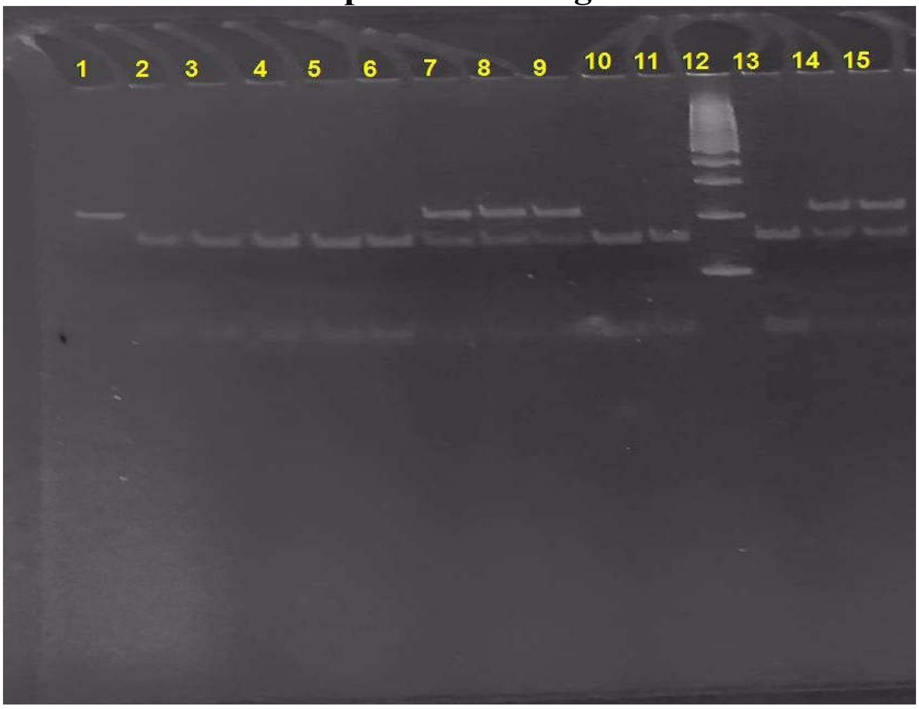

Fig. S8 Representative RFLP gel image of rs1048943 of *CYP1A1* for genotyping. The amplicons are 204 bp. Restriction digestion by BsrDI generates 3 distinct bands; 204 bp, 149 bp, and 55 bp for heterozygous genotype (AG). The homozygous wild type genotype (AA) gives 2 separate bands, 149 bp and 55 bp, whereas the homozygous variant genotype (GG) remains uncut with 204 bp. The unprocessed raw image was also provided.

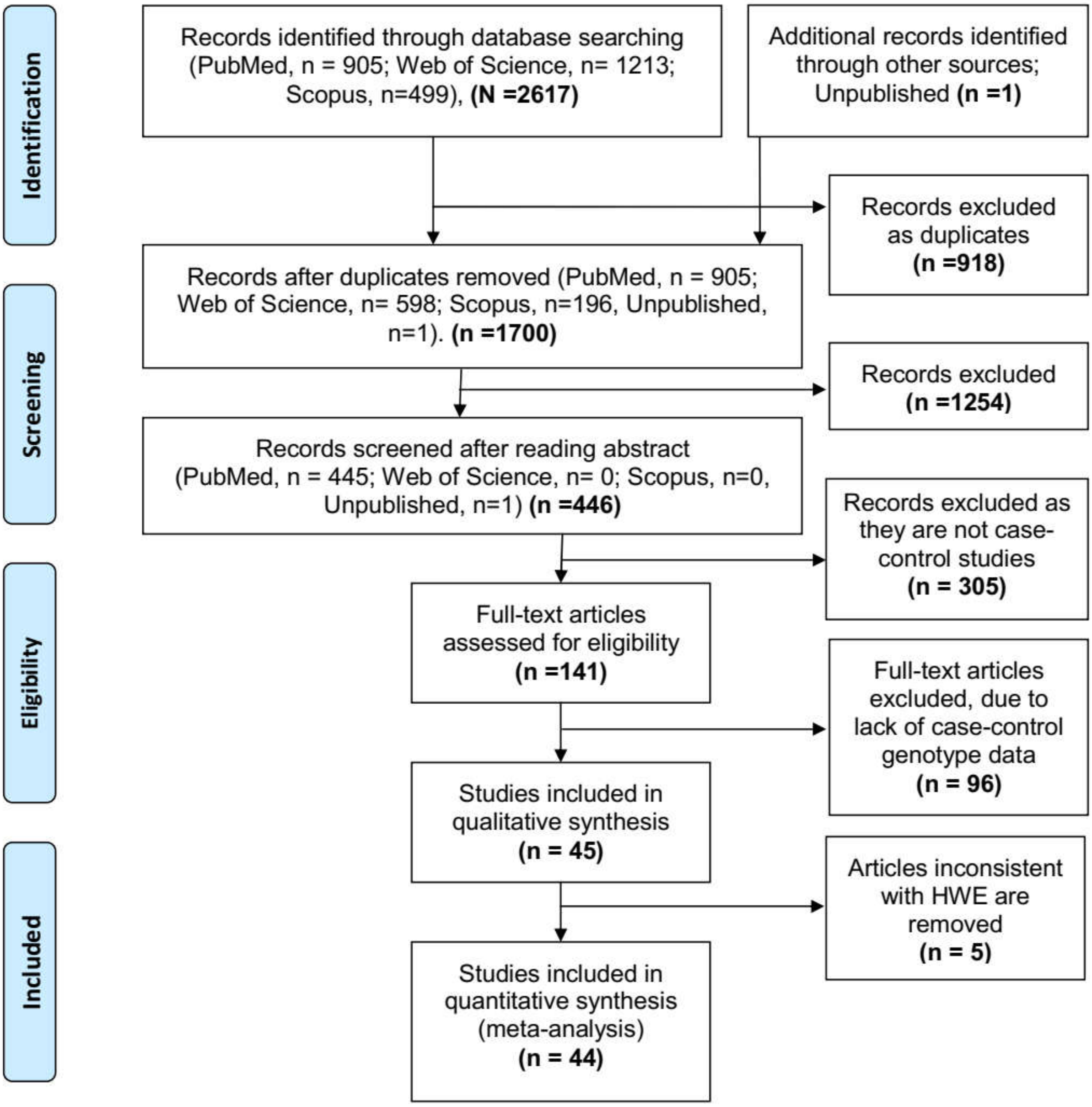

Fig. S9 The pathway of selecting candidate association studies for meta-analysis of rs1048943 (*CYP1A1*) on studies reported worldwide. The flow diagram is constructed as per the PRISMA guidelines [**Source:** Moher, D., Liberati, A., Tetzlaff, J., Altman, D. G. & Group, P. Preferred reporting items for systematic reviews and meta-analyses: the PRISMA statement. *BMJ* **339**, b2535, doi:10.1136/bmj.b2535 (2009)].

rs1048943/*CYP1A1*

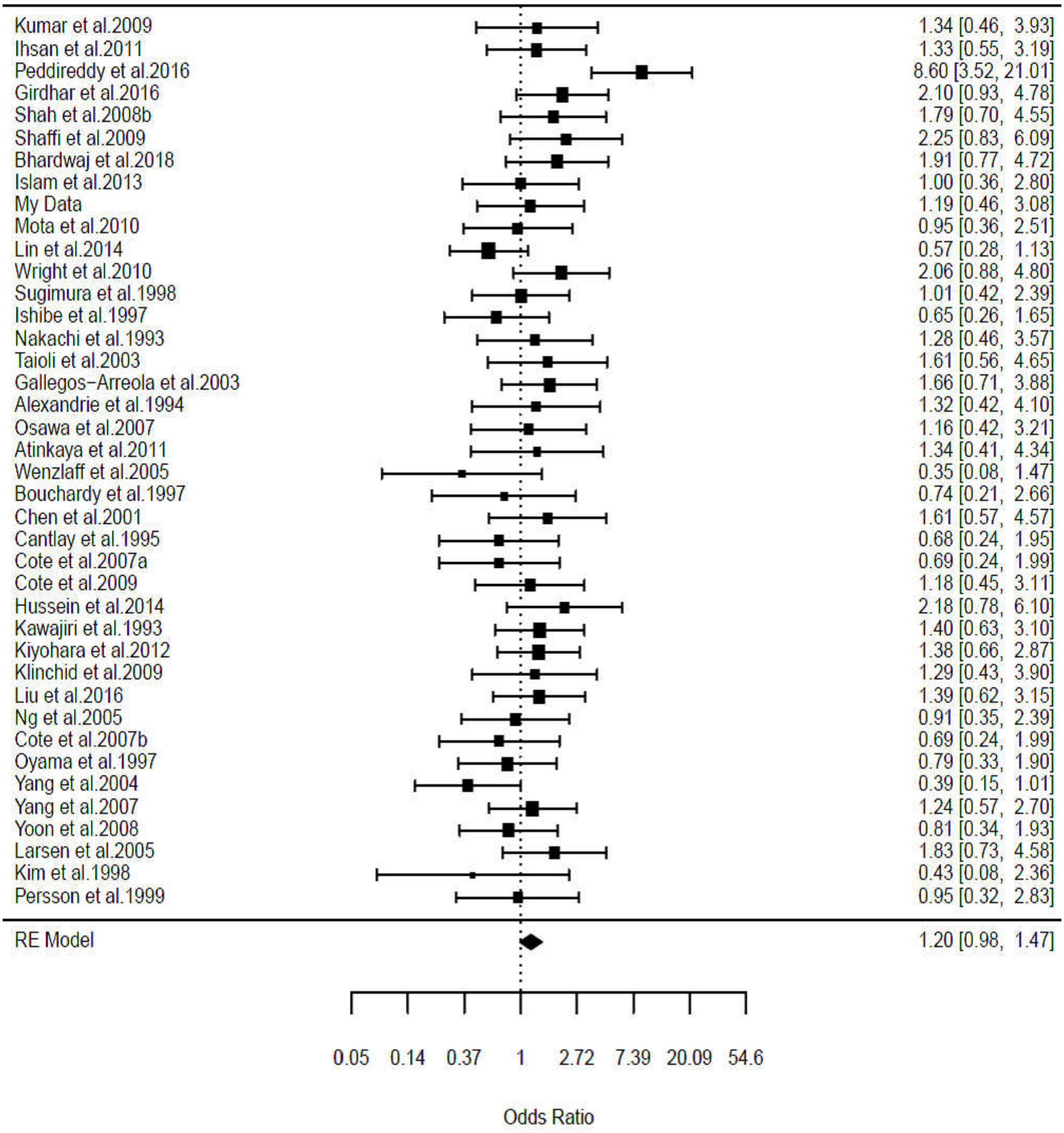

[A].

rs1048943/*CYP1A1*

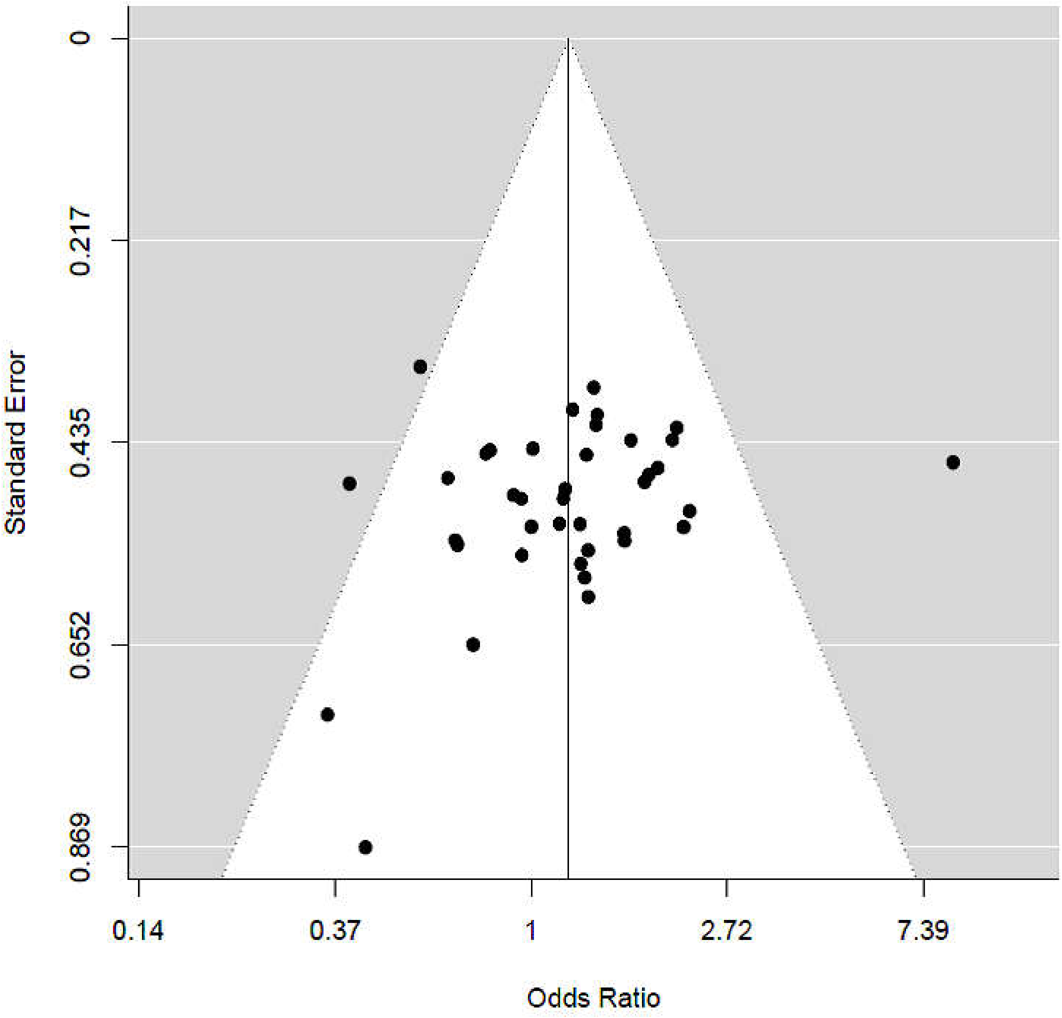

[B].

Fig. S10 Forest plots depicting the odds ratios (ORs), and 95% CI of the variant, **rs1048943/*CYP1A1*** for its association with overall lung cancer risk in the world population, **[A] in a random-effects model, [B] Funnel plot that** shows no evidence of publication bias between the studies reporting the polymorphism **rs1048943/*CYP1A1*.** *The above results were generated in a dominant genetic model.* **The forest plots of the significant associations (*p* < *0.05*) are given.** The figures were generated in the 'metafor' package (http://www.metafor-project.org) of R software (https://cran.r-project.org/)

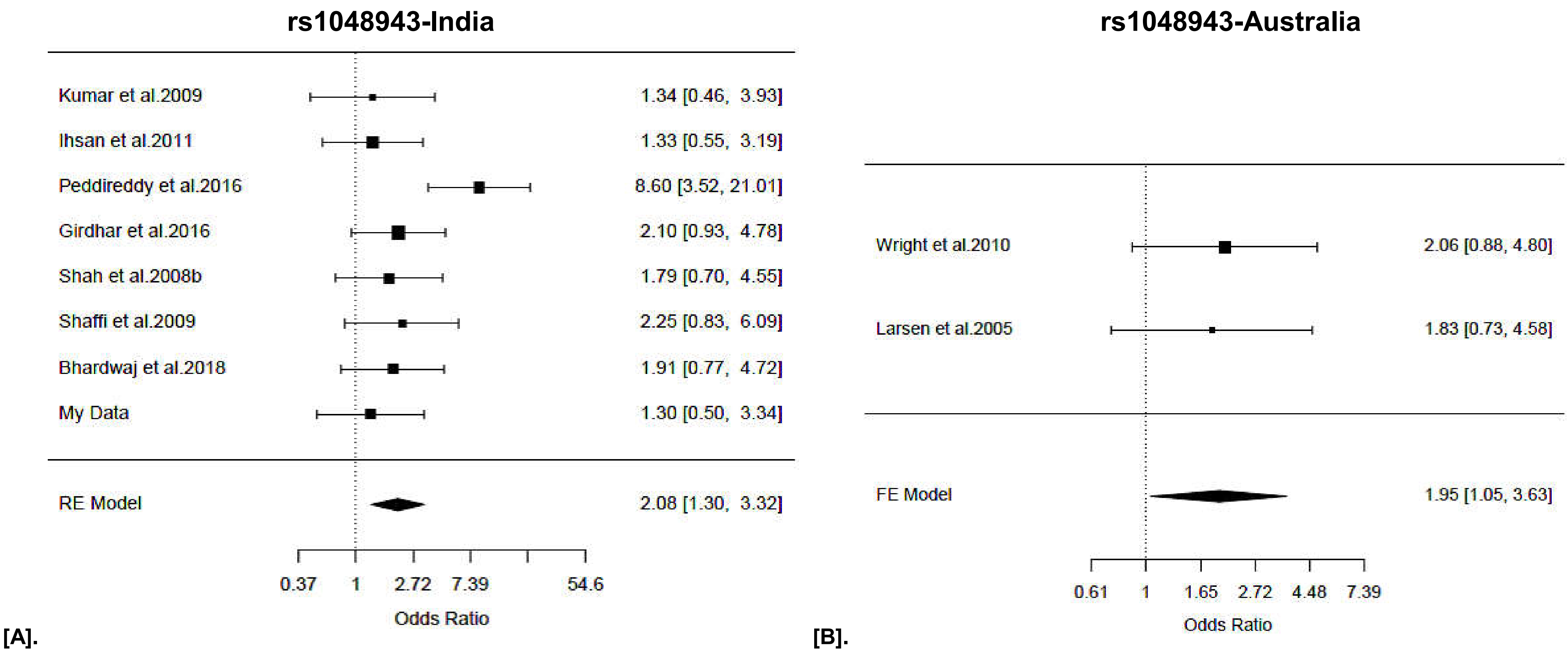

Fig. S11 Contrasting association of rs1048943/*CYP1A1* with lung cancer across diverse populations of the world. Forest plots depicting the association of the variant **rs1048943/*CYP1A1*** with lung cancer risk, **[A] in the Indian population, [B] in the Australian population.** *The above results were generated in a dominant genetic model.* **($p < 0.05$).** The figures were generated in the 'metafor' package (http://www.metafor-project.org) of R software (https://cran.r-project.org/)

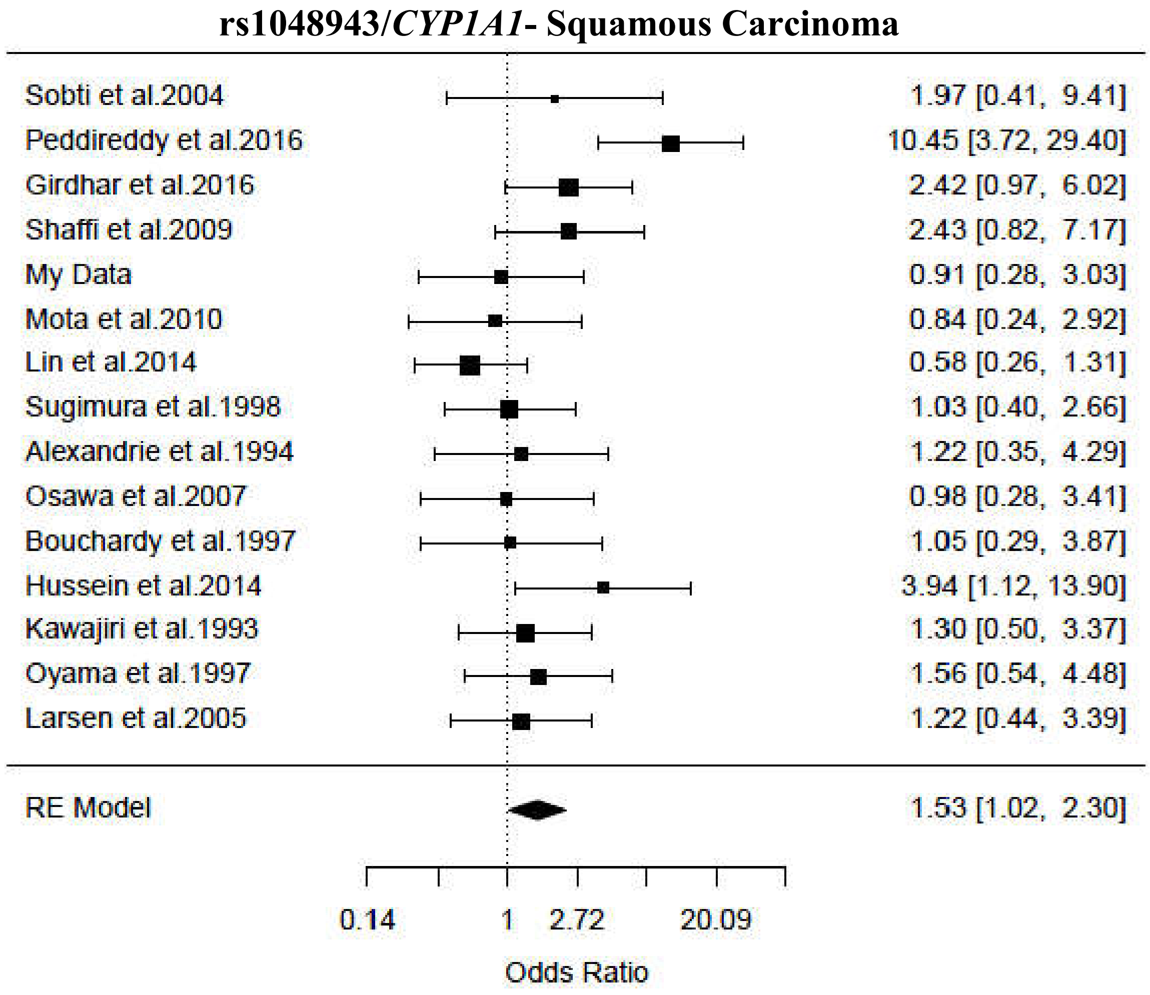
rs1048943/*CYP1A1*- Squamous Carcinoma
Sobti et al.2004
Peddireddy et al.2016
Girdhar et al.2016
Shaffi et al.2009
My Data
Mota et al.2010
Lin et al.2014
Sugimura et al.1998
Alexandrie et al.1994
Osawa et al.2007
Bouchardy et al.1997
Hussein et al.2014
Kawajiri et al.1993
Oyama et al.1997
Larsen et al.2005
RE Model
1.97 [0.41, 9.41]
10.45 [3.72, 29.40]
2.42 [0.97, 6.02]
2.43 [0.82, 7.17]
0.91 [0.28, 3.03]
0.84 [0.24, 2.92]
0.58 [0.26, 1.31]
1.03 [0.40, 2.66]
1.22 [0.35, 4.29]
0.98 [0.28, 3.41]
1.05 [0.29, 3.87]
3.94 [1.12, 13.90]
1.30 [0.50, 3.37]
1.56 [0.54, 4.48]
1.22 [0.44, 3.39]
1.53 [1.02, 2.30]
0.14
1
2.72
20.09
Odds Ratio

[A].

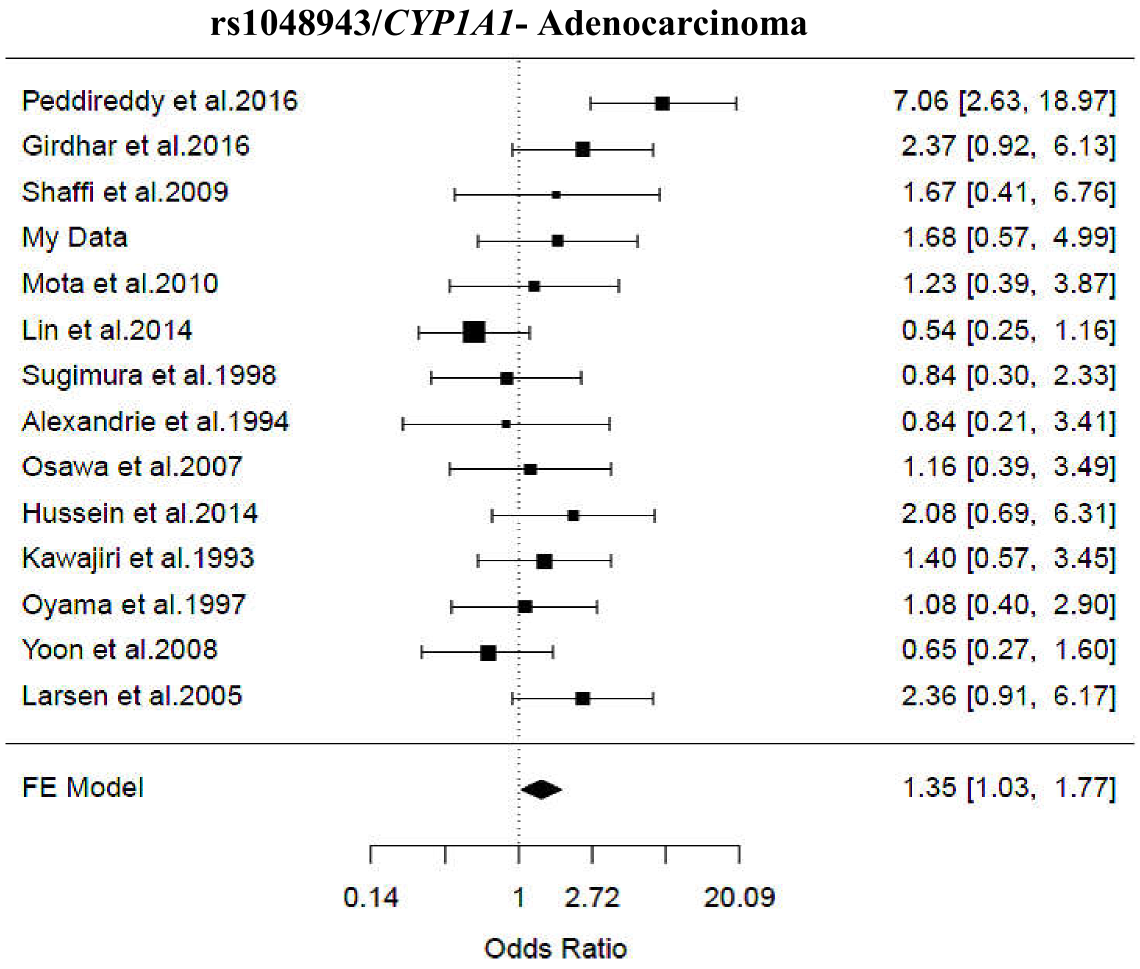
rs1048943/*CYP1A1*- Adenocarcinoma
Peddireddy et al.2016
Girdhar et al.2016
Shaffi et al.2009
My Data
Mota et al.2010
Lin et al.2014
Sugimura et al.1998
Alexandrie et al.1994
Osawa et al.2007
Hussein et al.2014
Kawajiri et al.1993
Oyama et al.1997
Yoon et al.2008
Larsen et al.2005
FE Model
7.06 [2.63, 18.97]
2.37 [0.92, 6.13]
1.67 [0.41, 6.76]
1.68 [0.57, 4.99]
1.23 [0.39, 3.87]
0.54 [0.25, 1.16]
0.84 [0.30, 2.33]
0.84 [0.21, 3.41]
1.16 [0.39, 3.49]
2.08 [0.69, 6.31]
1.40 [0.57, 3.45]
1.08 [0.40, 2.90]
0.65 [0.27, 1.60]
2.36 [0.91, 6.17]
1.35 [1.03, 1.77]
0.14
1
2.72
20.09
Odds Ratio

[B].

Fig. S12 [A] Forest plots depicting the odds ratios (ORs), and 95% CI of the variant, **rs1048943/*CYP1A1*** for its association with Squamous Carcinoma in the world population, in a random-effects model, **[B] Forest plots** depicting the odds ratios (ORs), and 95% CI of the variant, **rs1048943/*CYP1A1*** for its association with Adenocarcinoma in the world population, in a fixed-effect model. *The above results were generated in a dominant genetic model*. **The forest plots of the significant associations (p < *0.05*) are given.** The figures were generated in the 'metafor' package (http://www.metafor-project.org) of R software (https://cran.r-project.org/)

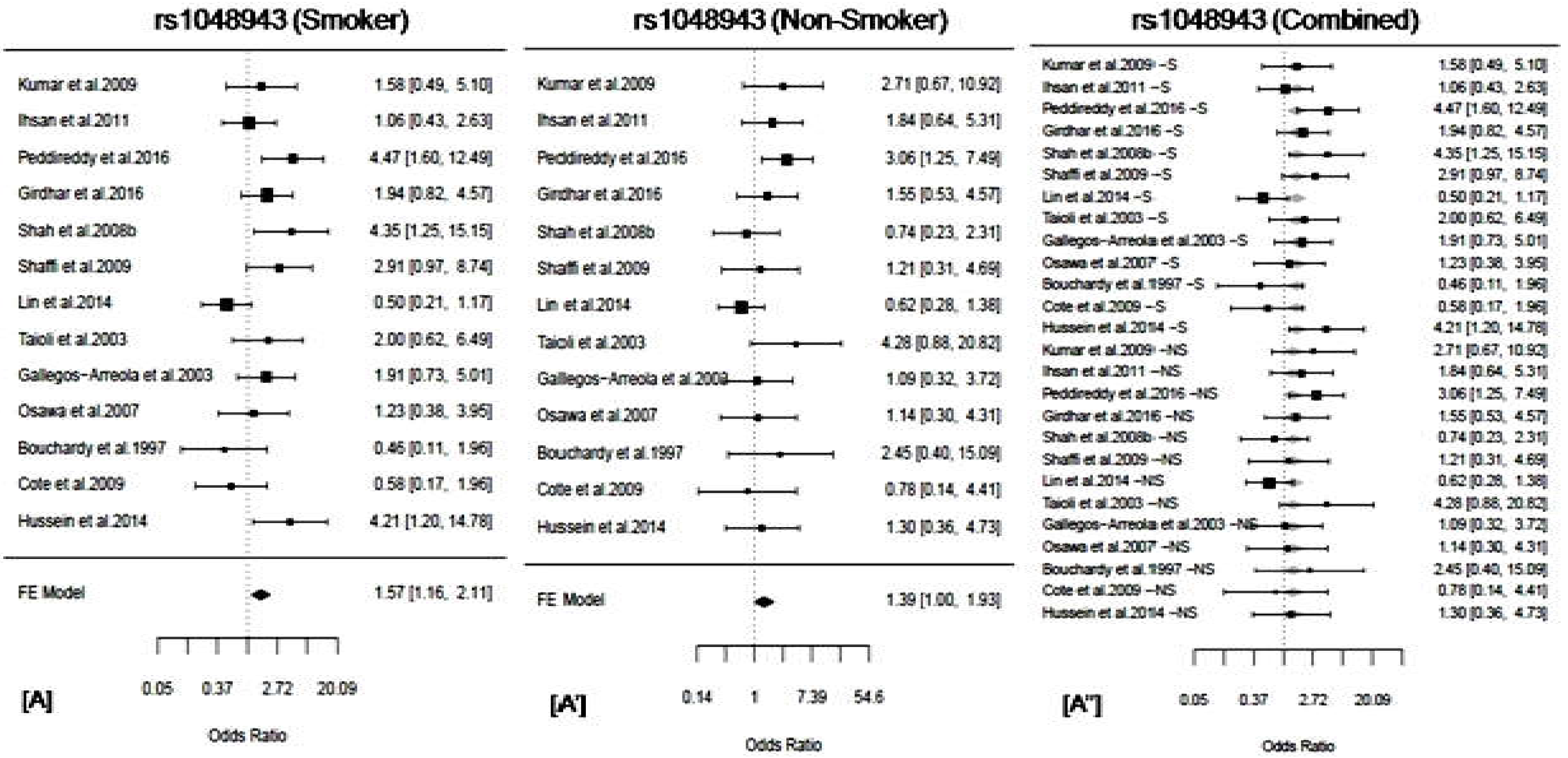

Fig. S13 [A] Forest plot depicting the odds ratios (ORs), and 95% CI of, **rs1048943/*CYP1A1*** for its association with lung cancer in smokers in a dominant model in the global population, **[A'] Forest plot** depicting the odds ratios (ORs), and 95% CI of, **rs1048943/*CYP1A1*** for its association with lung cancer in non-smokers in a dominant model in the global population, **[A''] Combine forest plot** depicting the odds ratios (ORs), and 95% CI of **rs1048943/*CYP1A1*** for its association with lung cancer stratified by smoking status in a dominant model in the global population. All the forest plots were generated in a fixed- effect meta-regression model assuming a common effect of the variant on lung cancer by smoking status. The figures were generated in the 'metafor' package (http://www.metafor-project.org) of R software (https://cran.r-project.org/)

Table S1. Summary data of 18 variants reported in the selected literature. Distribution of covariates, particularly tobacco smoking, histological status, and geographical region of the subjects, were recorded from all the 39 studies selected for meta-analysis that includes 18 variants from 11 genes for case-control data, Odds ratio, 95% Confidence Interval (CI).

Gene	Variant Identifiers	Genotypes	P value	OR (95% CI)	Lung Cancer Subtype		Mode of Genotyping	First Author-Year
	N/A	Present (+/+ or +/-)	N/A	N/A	N/A	N/A	PCR	Bag et al.2014
		Null (-/-)						
					Squamous cell	118	Multiplex PCR	Shukla et al. 2013a
	N/A	Present (+/+, +/-)	……	1 (Reference)	Adenocarcinoma	65		
					Mixed cell	27		
		Null (-/-)	0.002	1.87 (1.25-2.80)	Small cell	8		
	N/A	Present (+/+, +/-)	,…..	1 (Reference)	Squamous cell	96	PCR	Sobti et al.2008
					Adenocarcinoma	11		
					NSCLC	12		
		Null (-/-)	N/A	1.39 (0.74-2.62)	Small cell	24		
					NC	8		
					Squamous cell carcinoma	71	Multiplex PCR	Sobti et al.2004
Glutathione S transferase T1	N/A	Present (+/+, +/-)	…..	1 (Reference)	Small cell carcinoma	24		
		Null (-/-)	0.67	1.23 (0.52–2.87)	Adenocarcinoma	4		
					Large cell carcinoma	1		
					Squamous cell carcinoma	115	Multiplex PCR	Sharma et al.20115
	N/A	Present (+/+, +/-)	….	1 (Reference)	Adenocarcinoma	86		
		Null (-/-)	0.15	1.4 (0.87–2.2)				
					Small cell carcinoma	69		
	N/A	Present (+/+, +/-)	…..	1 (Reference)	Squamous cell carcinoma	21	Multiplex PCR-Sanger Sequencing	Kumar et al.2008
		Null (-/-)	N/A	1.3 (1.0–1.7)	Small cell carcinoma	13		
					Adenocarcinoma	35		
					Large cell carcinoma	6		
					Mixed cell tumors	18		
								Ishan et al.2014
					Not Reported	Not	Quantitative	

	N/A	(+/+),(+/-)	…..	1 (Reference)		Reported	real-time TaqMan PCR	
		(-/-)	0.01	0.65 (0.47-091)				
	N/A	Present (+/+, +/-)	…….	1 (Reference)	Not Reported	Not Reported	Multiplex PCR	Phukan et al.2014
		Null (-/-)	0.855	0.96 (0.64-1.44)				
	N/A	Present (+/+ or +/-)	…..	1 (Reference)	Not Reported	Not Reported		Ihsan et al. 2011
		Null (-/-)	0.06	0.62 (0.38–1.02)			PCR-RFLP	
	N/A		N/A	N/A	N/A	N/A	N/A	Sreeja et al. 2008
		Present (+/+)	N/A	N/A	N/A	N/A	N/A	
		Null (-/-)	N/A	N/A	N/A	N/A	N/A	
	N/A	Present (+/+)	……	1 (Reference)	Squamous-cell carcinoma	97	PCR	Peddireddy et al. 2016
		Null (-/-)	0.008	1.98 (1.18, 3.32)	Adenocarcinoma	109		
					Large cell and others	40		
	N/A	Present (+/+, +/-)	……	1 (Reference)	N/A	N/A	PCR-RFLP	Sreeja et al. 2005
		Null (-/-)	0.014	2.472				
	N/A	Present (+/+)	……	1 (Reference)	Squamous cell	118	PCR-RFLP	Shukla et al. 2013
		Null (-/-)	0.002	1.87 (1.25-2.80)	Adenocarcinoma	65		
					Mixed cell	27		
					Small cell	8		
	N/A	Present (+/+)	…..	……	SQCC	124	PCR-RFLP	Bhardwaja et al. 2018
		Null (-/-)	0.004	0.43 (0.28–0.66)	ADCC	95		
					SCLC	68		
					Others & Unknown	5		
	N/A	Present	....	1 (Ref)	N/A	N/A	PCR-RFLP	Masood et

								al.2016
		Null (-/-)	0.8	0.8 (0.2-3.3)				
Glutathione S Transferese Mu 1	N/A	(+/+)	….	1 (ref.)	N/A	N/A	PCR-RFLP	Ihsan et al. 2012
		(+/-)	0.37	0.73(0.37-1.44)				
		(-/-)	0.17	0.62(0.31-1.23)				
	N/A	Present (+/+, +/-)	……	1 (ref.)	N/A	N/A	PCR-RFLP	Ihsan et al. 2011
		Null (-/-)	0.8	0.95 (0.63–1.41)				
	N/A	Present	……	1 (ref.)	N/A	N/A	PCR-RFLP	Phukan et al. 2014
		Null	0.003	1.85 (1.24-2.76)				
	N/A	Present (+/+, +/-)	N/A	N/A	N/A	N/A	PCR-RFLP	Kumar et al. 2009
		Null (-/-)	N/A	N/A				
	N/A	Present (+/+, +/-)	…….	1 (ref.)	N/A	N/A	PCR-RFLP	Sobti et al. 2008
		Null (-/-)	N/A	1.30 (0.81 2.08)				
	N/A	Present (+/+, +/-)	……	1 (Ref.)	N/A	N/A		Shah et al. 2008
		Null (-/-)	0.001	2.26 (1.30–3.80)			PCR-RFLP	
	N/A	Present (+/+, +/-)	…….	1.0 (Ref.)	N/A	N/A	PCR-RFLP	Singh et al. 2011
		Null (-/-)	0.04	1.57 (1.02–2.40)				

	N/A	Present (+/+)	……	1(Ref.)	Squamous cell carcinoma	71	PCR-RFLP	Sobti et al. 2004
		Null (-/-)	0.46	1.22 (0.64–2.36)	Small cell carcinoma	24		
					Adenocarcinoma	4		
					Large cell carcinoma	1		
	N/A	Present (+/+, +/-)	…..	1(Ref.)	Squamous cell	118	PCR-RFLP	Shukla et al. 2013
		Null (-/-)	0.875	1.03 (0.71-1.51)	Adenocarcinoma	65		
					Mixed cell,Small cell	35		
	N/A	Present (+/+, +/-)	N/A	N/A	SQCC	115	PCR-RFLP	Sharma et al. 2015
		Null (-/-)	N/A	N/A	ADCC	86		
					SCLC	69		
	N/A	Present (+/+, +/-)	……	1 (Reference)	N/A	N/A	PCR-RFLP	Sreeja et al. 2005
		Null (-/-)	0.453	1.232(0.714–2.126)				
	N/A	Present (+/+, +/-)	N/A	N/A	N/A	N/A	N/A	Sreeja et al.2008
		Null (-/-)	N/A	N/A	N/A	N/A	N/A	
	N/A	Present (+/+, +/-)	…..	1 (Reference)	Squamous-cell carcinoma	97	PCR-RFLP	Peddireddy et al. 2016
					Adenocarcinoma	109		
		Null (-/-)	0.83	1.04 (0.69, 1.56)	Large cell and others	40		
					Squamous cell	118	PCR-RFLP	Shukla et al.2013b
					Adenocarcinoma	65		
	N/A	Present (+/+)	……	1 Reference	Mixed cell	27		
		Null(-/-)	0.875	1.03 (0.71-1.51)	Small cell	8		
					SQCC	124	PCR,RFLP	Bhardwaja et al. 2018
	N/A	Present (+/+)	…..	1 (Reference)	ADCC	95		
	N/A	Null(-/-)	< 0.0001	4.42 (2.86–6.83)	SCLC	68		
					Unknown,Others	5		
	N/A	Present (+/+)	....	1 (Reference)	N/A	N/A	PCR-RFLP	Masood et al.2016
	N/A	Null(-/-)	0.9	0.9 (0.4-2.3)				

Glutathione S Transferese P 1	rs1695	AA	N/A	N/A	N/A	N/A	N/A	Sreeja et al. 2008
		AG	N/A	N/A	N/A	N/A	N/A	
		GG	N/A	N/A	N/A	N/A	N/A	
	rs1695	AA	……	1 (Reference)	N/A	N/A	PCR,RFLP	Ihsan et al. 2011
		AG	0.07	1.46 (0.95–2.23)	N/A	N/A		
		GG	0.84	1.09 (0.43–2.77)	N/A	N/A		
	rs1695	AA	N/A	N/A	N/A	N/A	PCR,RFLP	Kumar et al. 2008
		AG	N/A	N/A	N/A	N/A		
		GG	N/A	N/A	N/A	N/A		
	rs1695	AA	…..	1 (Reference)	N/A	N/A	PCR-RFLP	Sobti et al. 2008
		AG	…..	0.63 (0.40 1.01)	N/A	N/A		
		GG	……	0.64 (0.19 2.22)	N/A	N/A		
					Non-small squamous	160	PCR-RFLP	Saikia et al. 2014
XRCC 1	rs25487	GG	…..	1(Ref.)	Non-small adenocarcinoma	65		
		GA	0.654	1.08 (0.78-1.48)	Small cell carcinoma	26		
		AA	0.289	1.37 (0.77-2.43)	Other	21		
	rs25487	GG	……	1(Ref.)	Adenocarcinoma	55	PCR-RFLP	Natukula et al. 2013
		AA	0.02*	2.613	Adenocarcinoma	29		
		GA	0.02	1.566	Undifferentiated	16		
	rs25487	GG	0.109	2.001 (0.858-4.670)	N/A	N/A	PCR-RFLP	Uppal et al. 2014
		GA	<0.001*	0.276(0.150-0.507)	N/A	N/A		
		AA	<0.001*	2.862 (1.504-5.447)	N/A	N/A		
	rs25487	GG	……	1(Ref.)			PCR-RFLP	Pachouri et al. 2007
		GA	0.05	0.3 (0.19–0.67)	SQCC	57		
		AA		0.4 (0.18–1.18)0	AC	25		
	rs1799782	CC	……	1(Ref.)	SCLC	11		
		CT	0.05	1.0 (0.75–01.45)	NSCLC	5		
		TT		1.3 (0.63–2.92)	PD	5		
	rs25487	GG	…..	1(Ref.)	NSCLC	171	PCR-RFLP	Sreeja et al. 2007
		GA	0.11	1.4 (0.920–2.148)	SCLC	20		

		AA	0.007	2.1 (1.224–3.669)	Unclassified	20		
	rs1799782	CC	…..	1(Ref.)				Singh et al. 2016
		CT	0.2	1.29 (0.86–1.95)			PCR-RFLP	
		TT	0.48	0.50(0.075–3.38)	ADCC	139		
	rs25487	GG	…..	1(Ref.)	SQCC	81		
		GA	0.5	0.88 (0.60–1.28)				
		AA	0.06	0.61 (0.37–1.00)	SCLC	4		
	rs915927	AA	….	1(Ref.)				
		AG	0.18	1.20 (0.89–1.70)				
		GG	0.82	0.93 (0.54–1.61)	Others	0		
	rs25487	GG	……	……			PCR-RFLP	Singh et al. 2017
		GA	0.82	……	ADCC	83		
		AA	0.54	……				
	rs1799782	CC	……	……				
		CT	0.9	……	SQCC	101		
		TT	……	……				
	rs915927	AA	……	……				
		AG	0.87	……	SCLC	55		
		GG	0.6	……				
	rs25487	GG	…..	1(Ref.)	SQCC	124	PCR-RFLP	Bhardwaj et al. 2017
	rs25487	GA	0.33	0.80 (0.52–1.24)				
	rs25487	AA	0.1	0.77 (0.58–1.01)				
					ADCC	95		
	rs1799782	CC	…..	1(Ref.)				
	rs1799782	CT	0.22	1.40 (0.87–2.26)				
	rs1799782	TT	0.78	0.80 (0.23–2.69)	SCLC	68		
	rs915927	AA	…..	1(Ref.)	Others	4		
	rs915927	AG	0.2	1.38 (0.91–2.09)				
	rs915927	GG	0.45	2.83 (0.59–1.18)	Unknown	1		

	rs1042522	GG	…..	……	NA	NA	PCR	Ihsan et al. 2011
		GC	0.71	1.11				
		CC	0.83	1.06				
					Squamous cell carcinoma	11	PCR	Jain et al. 2010
	rs1042522	GG	0.004	5.13	Adenocarcinoma	3		
		GC	0.18	……	Small cell carcinoma	4		
		CC	0.14	……	Large cell carcinoma	1		
	rs1042522	GG	…..	1(Ref.)	N/A	N/A	PCR-RFLP	Sobti et al. 2009
		GC	0.7	1.1 (0.63–2.04)				
		CC	0.5	1.3 (0.66–2.55)				
	rs1042522	GG	….	1(Ref.)	ADCC	125	PCR-RFLP	Kumari et al. 2016
		GC	0.82	1.04 (0.75–1.42)	SCLC	134		
		CC	0.96	0.99 (0.66–1.48)	SQCC	152		
					Others	9		
	rs1042522	GG	……	1(Ref.)	Adenocarcinoma	34	PCR-RFLP	Tilak et al. 2013
		GC	< 0.05	1.86a (1.05–3.27)	Squamous cell	102		
		CC	< 0.05	2.78a (1.31–6.03)	Others	39		
	rs1042522	GG	N/A		Adenocarcinoma	14	PCR-RFLP	Choudhury et al. 2015
		GC			Squamous cell	22		
		CC			SCLC	14		
CYP1A1								
	rs4646903	TT	…..	1.0 (Ref)	N/A	N/A	PCR	Ihsan et al.2011
		TC	0.01	1.69 (1.11–2.59)				
		CC	0.15	1.53 (0.84–2.78)				
	rs1048943	AA	……	1.0 (Ref)				
		AG	0.48	1.16 (0.75–1.80)				
		GG	0.13	2.18 (0.78–6.09)				
	rs1048943	AA	….	1.0 (Ref)				
		AG	…..	1.1 (0.8–1.3)	N/A	N/A	PCR,RFLP	Kumar et al.2009
		GG	…..	3.8 (0.1–28.8)				

	rs4646903	TT	…..	1.0 (Ref)	Squamous cell	118	PCR	Shukla et al.2013c
		TC	0.4	0.69 (0.29-1.64)	Adenocarcinoma	65		
		CC	0.1	0.50 (0.21-1.15)	Mixed cell	27		
					Small cell	8		
	rs4646903	CC	……	1.0 (Ref)	N/A	N/A	PCR-RFLP	Sreeja et al.2005
		CT	0.091	1.543				
		TT	0.004	3.597				
	rs4646903	TT	……	1.0 (Ref)	Squamous-cell carcinoma	97	PCR-RFLP	Peddireddy et al. 2016
		TC	0.42	1.06 (0.73, 1.55)	Adenocarcinoma	109		
		CC	0.007	2.25 (1.16, 4.37)				
					Large cell and others	40		
	rs1048943	AA	……	1.0 (Ref)				
		AG	0.001	8.82 (5.67, 13.72)				
		GG	0.12	1.59 (0.88, 2.89)				
					Squamous cell carcinoma	71	PCR-RFLP	Sobti et al. 2004
	rs4646903	CC	……	1.0 (Ref)	Small cell carcinoma	24		
		CT	Not Reported	1.16 (0.60–2.22)	Adenocarcinoma	4		
		TT	Not Reported	1.17 (0.32–4.23	Large cell carcinoma	1		
	rs4646903	TT	…..	1.00 (Ref.)	SCC	148	PCR-RFLP	Girdhar et al. 2016
		CT	0.27	1.19 (0.86-1.6)	ADC	117		
		CC	0.0001	3.15 (1.74-5.7)	SCLC	82		
					Unclassified	6		
	rs1048943	AA	……	1.00 (Ref.)				
		AG	<0.0001	2.32 (1.62-3.3)				
		GG	0.701	0.78 (0.22-2.7)				
	rs4646903	TT	……	1.0 (Ref.)	N/A	N/A	PCR-RFLP,DNA sequencing.	Shah et al.2008
		CT	0.26	1.34 (0.79–2.25)				
		CC						
	rs1048943	AA	…..	1.0 (Ref.)				
		AG	0.22	1.41 (0.80–2.47)				
		GG						
	rs1799814	CC	….	1.0 (Ref.)				
		CA	0.001	3.57 (1.65–7.69)				
		AA						

		TT	N/A	N/A				
	rs4646903	CT	N/A	N/A	SCC	69		
		CC	N/A	N/A				Shaffi et al. 2009
			N/A	N/A	AC	18	PCR-RFLP	
		AA	N/A	N/A				
	rs1048943	AG	N/A	N/A	Others	22		
		GG	N/A	N/A				
		TT	……	1.0 (Ref.)	SQCC	124		
	rs4646903	TC	0.32	1.24 (0.84–1.82)	ADCC	95		
		CC	0.02	1.61 (1.10–2.36)	SCLC	68		
					Others	4	PCR-RFLP	Bhardwaj et al. 2017
		AA	…….	1.0 (Ref.)	Unknown	1		
	rs1048943	AG	0.002	2.35 (1.51–3.66)				
		GG	0.83	1.07 (0.54–2.13)				
					Non-small squamous	160		
					Non-small adenocarcinoma	65		
	rs13181	AA	…..	1.0 (Ref)			PCR-RFLP	Saikia et al.2014
		AC	0.795	1.04 (0.76-1.43)	Small cell carcinoma	26		
		CC	0.352	1.26 (0.78-2.04)	Other	21		
ERCC2								
		AA	……	1.0 (Ref)	SQCC	135		
	rs13181	AC	0.81	1.03–1.43	ADCC	120	PCR-RFLP	Lawania et al.2017
		CC	0.01	1.96 (1.17–3.27)	SCLC	111		
					Others	4		
					Unknown	0		
		GG	……	1.00(Ref.)	SQCC	128		
DKK3	rs3206824	GA	0.47	1.14 (0.79–1.64)				
		AA	0.68	1.29 (0.38–4.39)	ADCC	97		
					SCLC	70		
		AA	…..	1.00(Ref.)			PCR-RFLP	Bahl et al.2017
DKK2	rs447372	AG	0.37	0.85 (0.60–1.20)	Others	4		
		GG	0.2	1.52 (0.78–2.95)				
					Unknown	1		
		CC	……	1.00(Ref.)				
DKK2	rs419558	CT	0.002	1.77 (1.21–2.58)				
		TT	……	……				

DKK2	rs17037102	GG	……	1.00(Ref.)				
		GA	0.01	1.62 (1.09–2.40)				
		AA	……	…….				
DKK3	rs3206824	GG	……	N/A			PCR-RFLP	Bahl et al.2018
		GA	0.66	N/A				
		AA	0.91	N/A				
					SQCC	86		
DKK2	rs447372	AA	……	N/A				
		AG	0.66	N/A	ADCC	72		
		GG	0.32	N/A				
					SCLC	51		
DKK2	rs17037102	GG	……	N/A				
		GA	0.76	N/A	Others	3		
		AA	……	N/A				
					Unknown	0		
DKK2	rs419558	CC	……	N/A				
		CT	0.76	N/A				
		TT	……	N/A				
DKK2	rs419558	CC	……	1.00(Ref.)				Bahl et al.2017
	rs419558	CT	0.021	1.82(1.21–2.68)	SQCC	124		
	rs419558	TT	…..	…….				
					ADCC	95		
DKK2	rs447372	AA	……	1.00(Ref.)			PCR-RFLP	
	rs447372	AG	0.65	0.88(0.62–1.26)	SCLC	68		
	rs447372	GG	0.18	1.58(0.80–3.15)				
					Others	4		
DKK2	rs17037102	GG	……	1.00(Ref.)				
	rs17037102	GA	0.04	1.73(1.14–2.62)	Unknown	1		
	rs17037102	AA	…..	……				
DKK3	rs3206824	GG	……	1.00(Ref.)				
	rs3206824	GA	0.58	1.17(0.80–1.71)				
	rs3206824	AA	0.5	1.61(0.43–5.95)				
AXIN2	rs2240308	CC	…….	1.00(Ref.)				Bahl et al.2017
		CT	0.49	0.87 (0.59–1.28)	SQCC	131		
		CT	0.01	0.55 (0.34–0.87)				
					ADCC	97		

		GG	……..	1.00(Ref.)				
	rs2240307	GA	0.002	0.45 (0.27–0.75)	SCLC	72		
		AA	0.9	1.08 (0.37–3.73)				
					Others	2		
		AA	…….	1.00(Ref.)			PCR-RFLP	
	rs35285779	AG	0.41	0.83 (0.53–1.29)	Unknown	1		
		GG	……	……				
		GG	……	1.00(Ref.)				
	rs9915936	GA	0.2	0.56 (0.23–1.35)				
		AA	……	…….				
		CC	…..	……				
	rs2240308	CT	0.76	……				
		TT	0.32	…….	SQCC	86		
		GG	…….	…….	ADCC	72		
	rs9915936	GA	0.76	…….				
		AA	0.32	…….	SCLC	51		
		AA	…….	…….	Others	3		Bahl et al.2018
	rs35285779	AG	0.76	…….			PCR-RFLP	
		GG	……	…….				
		TT	…….	…….				
	rs2240307	TC	0.76	…….				
		CC	…….	…….				
		CC	…….	1.00(Ref.)				
	rs2240308	CT	0.97	0.99(0.66–1.47)				
		TT	<0.0001	0.30(0.19–0.48)	SQCC	124		
		GG	…….	1.00(Ref.)	ADCC	95		
	rs9915936	GA	0.021	0.45(0.26–0.76)				Bahl et al.2017
		AA	0.4	0.96(0.28–3.35)	SCLC	68		
							PCR-RFLP	
		TT	…..	1.00(Ref.)				
	rs2240307	TC	0.46	0.57(0.23–1.41)				
		CC	…..	…..				

Table S2. The distribution of clinical and demographic parameters in the East Indian sample population.

Variables	Cases, N=101	Controls, N=413	*p-value*
**Age**			
Mean ± SD	58.91±12.41	66.08±7.77	*<0.001****
**Pack Years**			
Mean ± SD	66.92±34.95	59.29±37.47	*0.02**
**Gender**			
Male	78	413	*<0.001****
Female	23	0	
**Tumor Histology**			
Adenocarcinoma (ADCC)	49		
Squamous Cell Carcinoma (SQCC)	38		
Small Cell Lung Cancer (SCLC)	13		
Others	1		
**TNM Staging**			
I	2		
II	11		
III	46		
IV	40		
Unknown	2		

Abbreviations: SD = Standard Deviation, N = total number of case patients or control subjects. Controls lack females. *p-values* for sex were derived from Chi - square test; Student t-test was used for age and pack-years. All P-values are two- sided. $p < 0.05$ was considered statistically significant.

Table S3. Allelic and genotypic association of rs1048943/*CYP1A1* with lung cancer in smokers of the East Indian sample population.

Gene-Polymorphism	Genotypes/ Alleles	Smoker lung cancer cases; n=101 (%)	Healthy Smoker controls; n=413 (%)	Model	Comparisons	OR (95% CI)[a]	*p-value*[a]	Adjusted OR (95% CI)[b]	*p-value*[b]
				—	—	—	—	—	—
CYP1A1-rs1048943A>G	AA	64 (63.4)	286 (69.24)						
	AG	36 (35.6)	122 (29.5)	Dominant	(AG+GG) vs AA	1.3 (0.83-2.05)	*0.26*	1.45 (0.80-2..61)	*0.22*
	GG	1 (0.01)	5 (0.012)	—	—	—	—	—	—
	**Alleles**				**Alleles**				
	A	164 (81.2)	694 (84.02)		A	—			
	G	38 (18.8)	132 (15.9)		**G**	1.22 (0.79-1.84)	*0.34*	—	—

Fisher's exact test was done to determine allelic association with lung cancer, and multivariate logistic regression was done in dominant model to ascertain genotypic association with lung cancer. [a]Unadjusted association with crude odds ratio and 95% confidence interval and *p-value*. [b]Adjusted for age, pack-years, alcohol consumption, tobacco chewing, betel quid chewing, and asbestos exposure within males; CI: Confidence interval, OR: Odds ratio, Significance levels: $p<$ *0.001 '***', 0.01 '**', 0.05 '*'*. n= number of cases and controls.

Table S4. Association of rs1048943/*CYP1A1* with lung cancer in covariate-stratified subgroups assesses gene-covariate interaction modifying lung cancer risk in the East Indian population.

Sub-Groups	Dominant Model	
	**OR (95% CI)**	***p-value***
Pack-years (<47 Pack-years)	1.09 (0.59-1.99)	*0.78*
Pack-years (≥47 Pack-years)	1.7 (0.84-3.45)	*0.14*
Interaction	*1.56 (0.62-3.95)*	*0.35*
Age (<64 years)	1.78 (0.99-3.21)	*0.06*
Age (≥ 64 years)	0.77 (0.35-1.67)	*0.51*
Interaction	*0.43 (0.16-1.14)*	*0.09*
Alcoholic	1.89 (0.99-3.61)	*0.55*
Non-Alcoholic	0.96 (0.49-1.83)	*0.9*
Interaction	*1.97 (0.79-4.94)*	*0.15*
Tobacco (Chewer)	1.43 (0.83-2.45)	*0.2*
Tobacco (Non-Chewer)	1.07 (0.41-2.77)	*0.89*
Interaction	*1.34 (0.45-3.99)*	*0.61*
Betel-Quid (Chewer)	1.28 (0.74-2.23)	*0.38*
Betel-Quid (Non-Chewer)	1.24 (0.50-3.07)	*0.64*
Interaction	*1.03 (0.36-2.98)*	*0.95*
Asbestos (Exposed)	1.6 (0.65-3.99)	*0.31*
Asbestos (Not Exposed)	1.29 (0.76-2.20)	*0.35*
Interaction	*1.24 (0.43-3.57)*	*0.69*

The analysis was done by logistic regression in an additive and dominant model.
Significance levels: $p < 0.001$ '***', *0.01* '**', *0.05* '*'.

Table S5. Association of rs1048943/*CYP1A1* with the histological subtypes and TNM stages of lung cancer in the East Indian population.

Gene-Polymorphism	Genetic Model	Characteristics	Association Results					
		Tumour subtypes	OR (95% CI)#	*p-value*#	OR (95% CI)§	*p-value*§	OR (95% CI)†	*p-value*†
CYP1A1-rs1048943A>G	Dominant Model	Lung Adenocarcinoma	**1.99 (1.09-3.63)**	***0.024***	**2.97 (1.35-6.69)**	***0.007***	**2.08 (1.08-4.02)**	***0.03***
		Lung Squamous Cell Carcinoma	0.92 (0.44-1.91)	*0.82*	0.89 (0.39-1.89)	*0.78*	0.99 (0.45-2.07)	*0.99*
		Small Cell Lung Cancer	0.61 (0.17-2.34)	*0.46*	0.69 (0.15-2.42)	*0.59*	0.63 (0.14-2.18)	*0.51*
		**Tumour Stages**						
CYP1A1-rs1048943A>G	Dominant Model	Stage I	Data Insufficient					
		Stage II	2.7 (0.81-9.02)	*0.11*	1.82 (0.10-2.72)	*0.64*	1.57 (0.10-2.43)	*0.73*
		Stage III	0.89 (0.45-1.74)	*0.73*	0.82 (0.36-1.74)	*0.62*	0.83 (0.36-1.77)	*0.64*
		Stage IV	1.41 (0.71-2.77)	*0.32*	1.84 (0.86-3.85)	*0.11*	1.94 (0.90-4.12)	*0.08*

#Logistic regression was done to obtain the unadjusted Odds Ratio (OR), 95% Confidence Interval (CI) and *p-value* for the tumour characteristics on variant genotypes in the dominant model. §Adjusted for age within males. †Adjusted for age, pack-years of smoking and ethnicity within males Significance levels: $p < 0.001$ '***', 0.01 '**', 0.05 '*'. The **bold** fonts depict significant associations.

Table S6. Summary data of rs1048943 (*CYP1A1*) reported in the selected literature worldwide. Covariate specific case-control data, Odds ratio, 95% Confidence Interval (CI), particularly tobacco smoking, mean age, histological status, and geographical region, were recorded from all the 40 studies selected for meta-analysis.

Gene	Variant Identifiers	Number of Cases/ Controls	Sex of Cases/ Controls	Mean Age of Cases/ Controls	Smokers in Cases/ Controls	Country of Report	PMID	Genotypes	p-value	OR (95% CI)	Lung Cancer Subtype		Mode of Genotyping	First Author-Year
CYP1A1	rs1048943	188/209	145M,43F/ 159M,50F	60.41±10.58/ 57.19±10.75	132/139	India	22206016	AA	……	1.0 (Ref)	N/A	N/A	PCR-RFLP	Ihsan et al.2011
								AG	0.48	1.16 (0.75–1.80)				
								GG	0.13	2.18 (0.78–6.09)				
	rs1048943	93/253	81M,12F/ 203M,50F	42.6 ± 6.3/ 39.8 ± 5.4	81/101	India	19009239	AA	….	1.0 (Ref)	N/A	N/A	PCR-RFLP	Kumar et al.2009
								AG	…..	1.1 (0.8–1.3)				
								GG	…..	3.8 (0.1–28.8)				
	rs1048943	246/250	177M,69F/ 180M,70F	57.57 ± 10.19/ 58.06 ± 9.56	106/63	India	27090234	AA	……	1.0 (Ref)	SQCC<br>ADC<br>LCC & Others	97<br>109<br>40	PCR-RFLP	Peddireddy et al. 2016
								AG	0.001	8.82 (5.67, 13.72)				
								GG	0.12	1.59 (0.88, 2.89)				
	rs1048943	353/351	305M,48F/ 300M,51F	57.55 ± 10.69/ 52.84 ± 10.80	278/250	India	27396354	AA	……	1.00 (Ref.)	SQCC<br>ADCC<br>SCLC<br>Unknown	148<br>117<br>82<br>6	PCR-RFLP	Girdhar et al. 2016
								AG	<0.0001	2.32 (1.62-3.3)				
								GG	0.701	0.78 (0.22-2.7)				
	rs1048943	294/263	200M,0F/ 200M,0F	56.0±9.0/ 43.0±12.0	120/62	India	18082227	AA	…..	1.0 (Ref.)	N/A	N/A	PCR-RFLP & DNA sequencing.	Shah et al.2008 (b)
								AG	0.22	1.41 (0.80–2.47)				
								GG						
	rs1048943	190/248	87M,22F/ 95M,68F	52.7/53.2	84/98	India	19827888	AA	N/A	N/A	SQCC<br>ADC<br>Others	69<br>18<br>22	PCR-RFLP	Shaffi et al. 2009
								AG	N/A	N/A				
								GG	N/A	N/A				
	rs1048943	250/237	252M,40F/ 241M,22F	57.38 ± 10.74/ 53.23 ± 10.44	229/197	India	29412865	AA	…….	1.0 (Ref.)	SQCC<br>ADCC<br>SCLC<br>Others<br>Unknown	124<br>95<br>68<br>4<br>1	PCR-RFLP	Bhardwaj et al. 2017
								AG	0.002	2.35 (1.51–3.66)				
								GG	0.83	1.07 (0.54–2.13)				
	rs1048943	250/237	252M,40F/ 241M,22F	57.38 ± 10.74/ 53.23 ± 10.44	97/104	Bangladesh	23178447	AA	…….	1.0 (Ref.)	SQCC<br>ADCC<br>SCLC<br>LCC<br>ADCSQCC	46<br>37<br>20<br>2<br>1	PCR-RFLP	Islam et al. 2013
								AG	0.013	2.10 (1.17–3.76)				
								GG	p>0.05	3.25 (0.79–13.48)				
								AA	…….	1.0 (Ref.)	SQCC	38		

	rs1048943	101/413	78M,23F/ 412M,1F	58.91±12.41/ 66.08±7.77	101/413	India	N/A	AG	....	....	ADCC SCLC Unknown	49 13 1	PCR-RFLP	My Data
								GG	0.303	1.25 (0.82-1.92)				
	rs1048943	175/217	130M,45F/ 143M,74F	62.1±10.2/ 70.8±11.8	0/0	Portugal	20054510	AA	……	1 (Ref.)	SQCC ADCC SCLC Others	53 59 33 30	PCR-RFLP	Regateiro et al. 2010
								AG	0.838	1.1 (0.7-1.7)				
								GG						
	rs1048943	526/526	358M,168F/ 363M,163F	N/A	233/234	China (Han Chinese)	25343551	AA	……	1 (Ref.)	SQCC ADCC Others	189 271 66	PCR-RFLP	Lin et al. 2014
								AG	<0.0001	0.57 (0.44-0.75)				
								GG	0.003	0.54 (0.36-0.81)				
	rs1048943	1040/784	710M,330F/ 531M,253F	65.7±10/ 63.1±12.7	NA/278	Australia	19608585	AA	....	1 (Ref.)	SQCC ADCC Others	373 407 260	PCR-RFLP	Wright et al. 2010
								AG	<0.001	2.057 (1.77–2.39)				
								GG						
	rs1048943	247/185	247M,0F/ 185M,0F	67.8±9.1/ 62.1±11.2	167/51	Japan	9610791	AA	…..	1 (Ref.)	SQCC ADCC SCLC ADCCSQCC Others Unknown	122 78 28 11 3 5	PCR-Silver Staining	Sugimura et al. 1998
								AG	0.395	0.84 (0.57-1.25)				
								GG	0.013	3.01 (1.26-7.18)				
	rs1048943	109/137	NA	60.4±11.1/ 58.8 ±11.8	104/84	African-Americans	9419406	AA	......	1 (Ref.)	SQCC ADCC SCLC	NA	PCR-RFLP	Ishibe et al. 1997
								AG	......	......				
								GG	>0.05	0.72 (0.11-4.79)				
	rs1048943	62/158	NA	65.0±11.4/ 64.5 ±12.7	57/92	Mexican-Americans	9419406	AA	......	1 (Ref.)	SQCC ADCC SCLC	NA	PCR-RFLP	Ishibe et al. 1997
								AG	......	......				
								GG	>0.05	1.34 (0.63-2.84)				
	rs1048943	85/170	78M,7F/156M,14F	65.2 ±8.3/ 65.2 ±8.3	61/127	Japanese	8319207	AA	N/A	N/A	SQCC	85	Allele-specific PCR	Nakachi et al. 1993
								AG						
								GG						
	rs1048943	261/1452	174M,87F/ 998M,454F	34.8 ± 7.4/ 40.7 ± 4.5	90/347	GSEC study (pooled analysis-European)	12690010	AA	NA	1.0 (Ref.)	SQCC ADCC SCLC	NA	PCR-RFLP	Taioli et al. 2003
								AG	NA	1.6 (0.9–3.1)				
								GG	NA	2.3 (0.1–37.9)				
	rs1048943	222/248	135M,87F/ 132M,116F	60.68±11.01/ 46.17 ±15.42	190/110	Mexican	28207103	AA	0.008	0.60 (0.41-0.88)	SQCC ADCC SCLC	83 97 28	PCR-RFLP	Gallegos-Arreola et
								AG	0.9	0.96 (0.65-1.41)				

								GG	<0.001	4.74 (2.30-10.49)	Non-differentiated	14		al. 2008
	rs1048943	296/329	210M,86F/ 298M,31F	NA	NA	Sweden	7923570	AA	NA	NA	SQCC	107	Allele-specific PCR	Alexandrie et al. 1994
								AG	NA	NA	ADCC<br>SCLC	84 58		
								GG	NA	NA	Others	47		
	rs1048943	113/121	74M,37F, 2U/73M,48F	NA	79/61	Japan	17477782	AA	…..	1(Ref)	SQCC	35	PCR-RFLP	Osawa et al. 2007
								AG	0.525	1.20	ADCC	68		
	rs1048943	128/122	119M,9F/ 46M,73F	58.86 ± 8.02/ 57.22 ± 8.44	102/24	Turkey	22893352	AA	……	1(Ref)	SQCC<br>ADCC<br>SCLC	NA	PCR-RFLP	Atinkaya et al. 2012
								AG	0.383	1.78 (0.48–6.54)				
								GG	0.749	1.75 (0.06–52.63)				
	rs1048943	160/181	66M,94F/ 89M,92F	63.2 ±13.5/ 54.9±13.3	NA	USA	16051642	AA	……	1(Ref)	SQCC<br>ADCC	26<br>89	PCR-RFLP	Wenzlaff et al. 2005
								AG	0.07	0.41 (0.14–1.22)	SCLC<br>LCLC	11<br>12		
								GG	....	....	Other/unknown	22		
	rs1048943	150/171	NA	58.4 ± 9.9/ 55.0 ± 11.0	85/102	France	23889070	AA<br>AG<br>GG	NA	NA	SQCC<br>SCLC	98<br>52	NA	Bouchardy et al. 1997
	rs1048943	106/106	87M,19F/ 87M,19F	56.9 ± 10.0/ 53.6±12.6	59/61	China	11406420	AA	……	1(Ref)	SQCC<br>ADCC<br>Other	49<br>46<br>11	Allele-specific PCR	Chen et al. 2001
								AG	0.23	1.39 (0.75–2.59)				
								GG	0.03	4.02 (0.96–19.43)				
	rs1048943	129/281	NA	NA	NA	Scottish	16696009	AA	……	1(Ref)	SQCC<br>ADCC<br>SCLC	NA	PCR-RFLP	Cantlay et al. 1995
								AG	....	....				
								GG	>0.05	1.48 (0.83-2.61)				
	rs1048943	383/449	190M,193F/ 199M,250F	42.5/41.4	352/248	United States	17174438	AA	……	1(Ref)	SQCC	47	PCR-RFLP	Cote et al. 2006
								AG	Caucasians: 0.06<br>African-Americans: 0.29	Caucasians: 0.41 (0.19—0.90)<br>African-Americans: 2.00 (0.38—10.43)	ADCC<br>SCLC<br>LCLC<br>Non-small cell<br>Other	191<br>51<br>36<br>16<br>42		
								GG	....	....				
	rs1048943	504/527	504F/527F	59.7 ±9.2/ 59.0± 9.5	305/100	United States	19174490	AA	……	1(Ref)	SQCC<br>ADCC<br>SCLC	NA	PCR-RFLP and TaqMan assays	Cote et al. 2009
								AG<br>GG	0.25	0.77 (0.42–1.41)				

	rs1048943	110/110	75M,35F/ 67M,43F	58.1 ± 11.2/ 59.6 ± 8.2	78/42	Egypt	24613751	AA	……	1(Ref)	SQCC<br>ADCC<br>Other	38<br>62<br>10	PCR-RFLP	Hussein et al. 2014
								AG	0.01	2 (1.1–3.5)				
								GG	0.01	4.4(1.2–14.1)				
	rs1048943	282/358	NA	NA	NA	Japan	8103989	AA	NA	NA	SQCC<br>ADCC<br>Undifferentiated cell carcinoma	95<br>128<br>59	Allele-specific PCR	Kawajiri et al. 1993
								AG						
								GG						
	rs1048943	462/379	287M,175F/ 283M,96F	66.1/55.9	198/129	Japan	22525558	AA	…..	1.0 (ref)	SQCC<br>ADCC<br>SCLC	NA	Allele-specific PCR	Kiyohara et al. 2012
								AG	0.338	1.18 (0.84–1.66)				
								GG	0.001	2.86 (1.54–5.32)				
	rs1048943	91/82	58M,33F/ 38M,44F	59.8± 9.73/ 57.8 ± 13.39	73/28	Thailand	19963114	AA	NA	1.0 (ref)	SQCC<br>ADCC	36<br>55	PCR-RFLP	Klinchid et al. 2009
								AG		1.23 (0.60—2.55)				
								GG						
	rs1048943	308/253	192M,116F/145M,108F	51.0 ± 6.2/ 50.0 ± 5.7	206/113	China	27819744	AA	…..	1.0 (ref)	SQCC<br>ADCC<br>SCLC<br>LCLC	87<br>161<br>46<br>14	PCR-RFLP	Liu et al. 2016
								AG	>0.05	1.19 (0.84-1.66)				
								GG	<0.001	2.85 (1.54-5.32)				
	rs1048943	126/162	NA	62.0 ± 13.7/ 63.4 ± 12.3	NA	China	15953982	AA	…N/A…	1.0 (ref)	SQCC<br>ADCC<br>Other	14<br>85<br>19	PCR-RFLP	Ng et al. 2004
								AG		0.8(0.5–1.3)				
								GG		2.6(0.9–7.0)				
	rs1048943	123/622	NA	NA	NA	Japan	2152821	AA	N/A	N/A	SQCC<br>ADCC	52<br>71	PCR-RFLP	Oyama et al. 1997
								AG						
								GG						
	rs1048943	200/144	NA	NA	111/54	China	15363546	AA	…..	1.0 (ref)	SQCC<br>ADCC<br>SCLC	NA	TaqMan assay	Yang et al. 2004
								AG	NA	2.68 (1.68–4.29)				
								GG	NA	1.71 (0.64–4.61)				
	rs1048943	318/353	215M,103F/ 218M,135F	55.4±9.6/48.3±9.6	137/124	Korea	17428572	AA	NA	NA	SQCC<br>ADCC<br>SCLC	71<br>174<br>31	PCR TaqMan assay	Yang et al. 2007
								AG	NA	NA				
								GG	NA	NA				

	rs1048943	213/213	NA	NA	NA	Korea	17980933	AA	……	1.0 (ref)	SQCC<br>ADCC<br>SCLC<br>Other	7<br>173<br>10<br>23	PCR TaqMan assay	Yoon et al. 2008
								AG	0.12	0.69 (0.43—1.10)				
								GG	0.64	0.79 (0.28—2.17)				
	rs1048943	1050/581	760M,290F/ 396M,185F	63.3 ±9.4/ 62.7 ±12.9	881/329	Australia	15986113	AA	NA	NA	SQCC<br>ADCC<br>Other	479<br>479<br>92	PCR-RFLP	Larsen et al. 2004
								AG	0.005	1.9 (1.2—2.9)				
								GG						
	rs1048943	17/48	NA	NA	NA	Korea	10102576	AA	NA	NA	SQCC<br>ADCC<br>SCLC	NA	PCR-RFLP	Kim et al. 1998
								AG	NA	NA				
								GG	NA	NA				
	rs1048943	76/122	42M,34F/ NA,NA	NA	20/NA	China	N/A	AA	NA	1.0 (ref)	SQCC<br>ADCC<br>SCLC<br>SQAD<br>PL	14<br>38<br>18<br>4<br>2	PCR-RFLP	Persson et al.1999
								AG	NA	0.83 (0.44–1.59)				
								GG	NA	1.81 (0.58–5.71)				

Table S7. A comprehensive list of meta-analysis results showing the overall association of rs1048943 (*CYP1A1*) with lung cancer worldwide, with a crude odds ratio (OR), 95% Confidence Interval (CI), p_{FDR}, Benjamini-Hochberg False Discovery Rate (FDR) corrected *p-value*, Heterogeneity indices H^2, I^2. Both the Genetic model and model used for meta-analysis are also mentioned. The results of both the Fixed-effect and Random-effects model were depicted.

			**Test for Association**				**Heterogeneity**		
**Gene Symbol**	**Variant ID**	**Number**	**Genetic Model**	**Comparison**	**Model**	**OR (95% CI);** ***p-value***	H^2	p_{Het}	I^2
CYP1A1	rs1048943	40	Dominant	**(AG+GG) vs AA**	**Fixed**	**1.21 (1.04-1.41);** ***0.01***	1.74	***0.08***	42.67
				(AG+GG) vs AA	Random	1.20 (0.98-1.47); *0.07*			

p-value<0.05, p_{FDR}<0.1, OR, Crude Odds Ratio, 95% CI, 95% Confidence Interval, I^2 and H^2 are measures of heterogeneity

p_{Het}<0.1 (Cochran's Q test), Significant associations are depicted in ***bold.*** *N/A stands for Not Applicable.*

Table S8. Association of rs1048943/*CYP1A1* with lung cancer stratified by the reported studies country and pooled into respective continents.

				**Test of association**			**Heterogeneity**		
**Variant (Gene)**	**Genetic Model**	**Sample Population**	**Number of Studies**	**Model**	**OR (95% CI)**	***p-value***	I^2	H^2	p_{Het}
rs1048943 (*CYP1A1*)	Dominant	**Overall**	**37**	**Fixed**	**1.21 (1.04-1.41)**	***0.01***	**42.67**	**1.74**	***0.08***
				Random	1.20 (0.98-1.47)	*0.07*			
		India	8	**Fixed**	**2.10 (1.52—2.91)**	***0.000007***	51.06	2.04	***0.09***
				**Random**	**2.08 (1.30—3.32)**	***0.002***			
		China	6	Fixed	0.83 (0.58—1.19)	*0.3*	41.27	1.7	*0.24*
		Japan	6	Fixed	1.16 (0.82—1.66)	*0.4*	4.11	1.04	*0.94*
		Korea	3	Fixed	0.93 (0.54—1.62)	*0.81*	23.99	1.32	*0.49*
		Europe	6	Fixed	1.05 (0.67—1.65)	*0.82*	8.6	1.09	*0.86*
		North America	6	Fixed	0.87 (0.58—1.32)	*0.52*	31.59	1.46	*0.42*
		Australia	2	**Fixed**	**1.95 (1.05—3.63)**	***0.04***	0.06	1	*0.85*

p-value<0.05, OR, Crude Odds Ratio, 95% CI, 95% Confidence Interval, I^2 and H^2 are measures of heterogeneity p_{Het}<0.1 (Cochran's Q test). Significant associations are depicted in bold. N/A stands for Not Applicable.

Table S9 Association of rs1048943/*CYP1A1* with lung cancer stratified by histological subtypes in the world population.

Variant ID/Gene	Histological subtypes	Test for Association				Heterogeneity		
		Number	Genetic Model	Model	OR (95% CI); *p-value*	I^2	H^2	p_{Het}
rs1048943/ *CYP1A1*	Squamous cell carcinoma	15	Dominant (AG+GG) vs AA	**Fixed**	**1.50 (1.14-1.99); *0.004***	51.33	2.05	***0.02***
				**Random**	**1.53 (1.02-2.30); *0.04***			
	Adenocarcinoma	14		**Fixed**	**1.35 (1.03-1.77); *0.028***	49.55	1.98	***0.032***
				Random	1.39 (0.95-2.05); *0.09*			
	Small Cell Lung Carcinoma	7		Fixed	0.94 (0.56-1.57); *0.79*	48.27	1.93	*0.2*

p-value<0.05, OR, Crude Odds Ratio, 95% CI, 95% Confidence Interval, I^2 and H^2 are measures of heterogeneity. p_{Het}<0.1 (Cochran's Q test). *Significant associations are depicted in **bold**.*

Table S10. Subgroup meta-analysis of rs1048943/*CYP1A1* with lung cancer; stratified by smoking status, i.e. in smokers and non-smokers. *OR, odds ratio (OR); 95% CI and heterogeneity indices, I^2, H^2.*

Variant ID/Gene Symbol	Sub-groups	Number of Studies	Genetic Model	OR (95% CI); *p-value*	I^2	H^2	*pHet*
rs1048943/ *CYP1A1*	**Smoker**	**13**	Dominant (AG+GG) vs AA	**1.57 (1.16-2.11); *0.003***	57.14	2.33	*0.22*
	Non- Smoker	13		1.39 (0.99-1.93); *0.051*	29.54	1.42	*0.42*

*p-value<0.05, OR, Crude Odds Ratio, 95% CI, 95% Confidence Interval, I^2 and H^2 are heterogeneity measures, p_{Het}<0.1 (Cochran's Q test). Significant associations are depicted in **bold**.*

Table S11. The results of the test for effect modification of rs1048943/*CYP1A1* on lung cancer by smoking.

**Moderator variable**	**Number of Studies**	**Genetic Model**	**Moderator Effect Size (θ)**	**θ low**	**θ high**	***p-value***
rs1048943/ *CYP1A1*	26	Dominant (AG+GG) vs AA	0.12	-0.32	0.56	*0.59*

p-value<0.05, Moderator effect size/interaction effect size (θ) is the change of effect sizes from non-smokers to smokers.
